# Targeting mitocytosis potentiates mitochondria drug delivery for antimetastasis therapy

**DOI:** 10.1126/sciadv.aec7150

**Published:** 2026-04-10

**Authors:** Yudi Deng, Yunhui Zhang, Fuya Jia, Peihang Jiang, Lian Li, Yuan Huang

**Affiliations:** Key Laboratory of Drug-Targeting and Drug Delivery System of the Education Ministry and Sichuan Province, Sichuan Engineering Laboratory for Plant-Sourced Drug and Sichuan Research Center for Drug Precision Industrial Technology, West China School of Pharmacy, Sichuan University, Chengdu 610041, China.

## Abstract

Mitocytosis is a compensatory pathway responding to mitochondria stress in migratory cells, which expels damaged mitochondria through migrasomes, preserving mitochondrial homeostasis and cellular viability. We found distinct responses to mitochondria-targeted therapy across breast tumor models distinguished by migrasome expression (4T1 > E0771 > EMT6). The antimetastatic efficacy of mitochondrial damage was notably compromised in the migrasome-high 4T1 tumor model due to robust mitocytosis activation, which is merely explored and lacks effective strategy. Here, we developed a mitochondria-targeted nanoplatform (RH-NPs) with the functions of mitocytosis inhibition and mitochondrial damage. Mitochondria-targeted triphenylphosphonium-modified lonidamine (TPP-LND) and integrin inhibitor cilengitide (CGT) were separately loaded into a nanodelivery system (TL/RH-NPs and CGT/RH-NPs, respectively). TL/RH-NPs effectively targeted and damaged tumor mitochondria. Simultaneously, upon mitocytosis activation, CGT/RH-NPs hitchhiked with damaged mitochondria into migrasomes to block mitocytosis via integrin inhibition. This strategy significantly potentiated antimetastatic efficacy in 4T1 tumor models, which established an effective approach for mitocytosis modulation and optimization of mitochondria-targeted therapies.

## Teaser

Targeted modulation of compensatory mitocytosis potentiated antitumor metastatic efficiency of mitochondrial damage.

## INTRODUCTION

Recent studies have identified migrasomes, newly found organelles formed in highly migratory cells through plasma membrane budding (0.5 to 3 μm) at the ends of retraction fibers during migration (*1*). It is reported that migrasomes are closely related to cell migration and have been shown to facilitate osteosarcoma metastasis (*2*). Further investigation uncovered mitocytosis, a migrasome-mediated process in migratory cells under mitochondrial stress characterized by increased energy demands and reactive oxygen species (ROS), which expels damaged mitochondria via migrasomes to maintain mitochondrial homeostasis and preserve cellular viability (*3*). Our study found that in three breast tumor models with different metastatic potential, the therapeutic efficacy of mitochondria-targeted therapy varied significantly. Especially, in 4T1 tumor model with the highest migrasome expression, the therapeutic efficacy was significantly compromised. Further investigation revealed that the significant activation of mitocytosis might be the reason for diminished therapeutic effects of mitochondria-targeted therapies. However, the therapeutic potential of mitocytosis modulation in highly migratory tumor cells remains unexplored. Therefore, we seek to enhance antimetastatic effects of mitochondria-targeted therapy via inhibiting mitocytosis, yet we are facing a critical barrier: the absence of effective regulatory tools for this process.

Mitochondria, as centers for aerobic respiration and cellular metabolism, play crucial roles in tumor progression (*4*, *5*), which are also key components in mitocytosis (*3*). Although directly targeting migrasomes remains extremely difficult, exploiting mitochondria as mediators—first targeting mitochondria and then hijacking them to reach migrasomes—presents a feasible strategy for mitocytosis modulation. However, targeting to mitochondria is also challenging for multiple physiological barriers and low permeability of mitochondrial membranes (*6*, *7*). Our earlier work revealed that tumor membrane–coated nanoparticles (T-NPs) enter tumor cells via the SNARE protein–mediated endoplasmic reticulum (ER)–Golgi apparatus (Golgi) pathway, with exocytosis inhibitors enabling ER-Golgi retention (*8*). This highlights their potential for subcellular organelle–targeted delivery, especially challenging mitochondria targeting. However, the following challenges arise: (i) T-NPs achieve tumor targeting but fail to reach mitochondria due to lack of mitochondria-targeting proteins and lysosome-escaping capabilities, and (ii) mitochondrial membrane–coated nanoparticles achieve mitochondrial targeting via membrane fusion but demonstrate poor tumor accumulation. These limitations restrict the further application of these two biomembranes in subcellular organelle–targeted drug delivery. Notably, hybrid membranes integrating dual biological functions offer a promising strategy (*9*). We therefore propose to engineer a hybrid membrane–coated delivery system with dual targeting capabilities of homologous tumor targeting and mitochondria targeting.

**Fig. 1.**
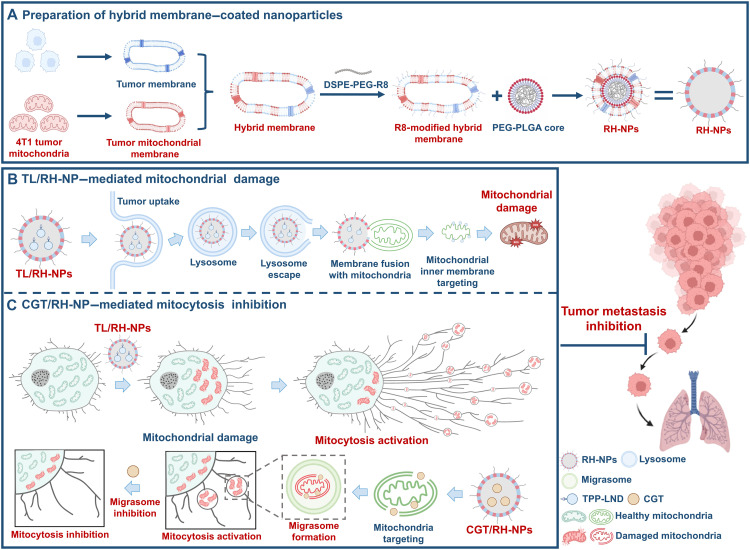
Illustration of synchronized mitochondrial damage and mitocytosis inhibition for potentiated antimetastatic efficiency. (**A**) Preparation of mitochondria-targeting nanoparticles coated with R8-modified hybrid membrane (RH-NPs). (**B**) TPP-LND loaded mitochondria-targeting nanoparticles (TL/RH-NPs) escape from lysosome degradation, fuse with the mitochondrial outer membrane, and deliver TPP-LND to the mitochondrial inner membrane, inducing mitochondrial damage. (**C**) Mitochondrial dysfunction triggers the compensatory mitocytosis pathway. Concurrently, CGT-loaded nanoparticles (CGT/RH-NPs) inhibit migrasome formation by colocalizing with damaged mitochondria and “hitchhike” into migrasomes, thereby suppressing migrasome formation and mitocytosis. Created in BioRender. Mahaha, M. (2026) https://BioRender.com/p3syvmx.

Here, we seek to potentiate the antimetastatic efficiency of mitochondria-targeted therapy incorporating mitocytosis inhibition and mitochondrial damage. Our design is realized by three key steps: (i) The mitochondria-targeted delivery system is developed with a hybrid membrane composed of homologous tumor cell membranes and membrane-fusing mitochondrial membranes. (ii) This system targets mitochondria, triggering mitochondrial damage and compensatory mitocytosis. (iii) The mitocytosis process is in turn inhibited via mitochondria-targeted system, which “hitchhikes” into migrasomes. Specifically, the mitochondria-targeted delivery system is further developed with lysosome escaping (*10*) R8 (RRRRRRRR) and drug-loaded polyethylene glycol (PEG)–poly-(lactic-*co*-glycolic acid) (PLGA) cores (RH-NPs; Fig. 1A). To induce mitochondrial damage, lonidamine (*11*, *12*) is directed to the inner mitochondrial membrane (IMM) by chemical incorporation with mitochondrial-targeting ligand triphenylphosphonium (TPP-LND) and encapsulated in RH-NPs (TL/RH-NPs). To inhibit mitocytosis, the integrin inhibitor cilengitide (CGT) (*13*) is encapsulated into RH-NPs (CGT/RH-NPs), leveraging the critical role of integrins in migrasome formation (*14*, *15*). We assume that TL/RH-NPs are actively internalized by tumor cells and fuse with the outer mitochondrial membrane to facilitate the interactions between positively charged TPP-LND and negatively charged IMM, thereby damaging mitochondria and exerting antimetastatic effects. Concurrently, CGT/RH-NPs colocalize with damaged mitochondria and hitchhike into migrasomes via the mitocytosis process activated by TL/RH-NPs, in turn suppressing this compensatory pathway (Fig. 1B). This design coordinates mitochondrial disruption with mitocytosis inhibition to block tumor metastasis, thereby providing an advantageous framework to modulate mitocytosis and potentiate the antimetastatic effects of mitochondria-targeted therapy.

## RESULTS

### Preparation and characterization of mitochondria-damaging hybrid membrane–coated nanoparticles

**Fig. 2.**
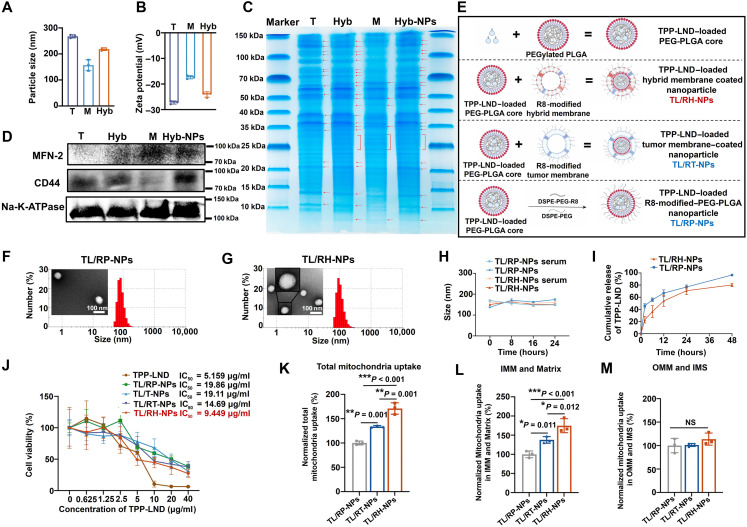
Preparation and characterization of mitochondria-damaging hybrid membrane–coated nanoparticles. (**A** and **B**) Particle size (A) and zeta potential (B) of tumor cell membrane (T), mitochondrial membrane (M), and hybrid membrane (Hyb; tumor cell membrane:mitochondrial membrane = 1:1). (**C**) SDS-PAGE of T, M, Hyb, and hybrid membrane–coated nanoparticles (Hyb-NPs). (**D**) WB analysis of mitochondrial membrane characteristic protein (MFN-2) and tumor membrane characteristic protein (CD44). (**E**) Illustration of the preparation of various PEG-modified or membrane-coated nanoparticles. Created in BioRender. Mahaha, M. (2026) https://BioRender.com/s1en2tw. (**F** and **G**) Particle size distribution and TEM images of TL/RP-NPs (F) and TL/RH-NPs (G). (**H**) Stability of TL/RP-NPs and TL/RH-NPs at 37°C within 24 hours in PBS and 10% serum. (**I**) Cumulative release of TPP-LND from TL/RP-NPs or TL/RH-NPs at 37°C within 24 hours in PBS. (**J**) 4T1 cell viability after treatment with TPP-LND, TL/RP-NPs, TL/T-NPs, TL/RT-NPs, or TL/RH-NPs for 24 hours. (**K** to **M**) Mitochondria uptake of TL/RP-NPs, TL/RT-NPs, or TL/RH-NPs in whole mitochondria (K), IMM plus Matrix (L), and outer mitochondrial membrane (OMM) plus intermembrane space (IMS) (M). All results were presented as means ± SD, *n* = 3. NS indicated no significance. T, tumor cell membrane; M, mitochondrial membrane; Hyb, hybrid membrane.

We first extracted and isolated tumor cell membrane and mitochondrial membrane and fused them to engineer hybrid membrane (Hyb) (at the mass ratio of T:M = 1:1). As shown in Fig. 2 (A and B), the particle size and the zeta potential of Hyb laid between T and M. Moreover, T and M showed apparently different expression of protein types in SDS–polyacrylamide gel electrophoresis (SDS-PAGE) (Fig. 2C). However, Hyb and Hyb-coated nanoparticles (Hyb-NPs) shared the same distribution of proteins, including all proteins from both T and M. In addition, Western blot (WB) analysis (Fig. 2D) assessed tumor characteristic proteins cell adhesion molecule 44 (CD44) and mitochondria membrane fusion protein 2 (MFN-2). Membrane protein Na–K–adenosine triphosphatase (ATPase) was used as internal control. Although T expressed a small amount of MFN-2, an obvious increase in the expression of MFN-2 was observed in Hyb, M, and Hyb-NPs, indicating their membrane fusion capability. The expression of tumor characteristic protein CD44 showed an opposite expression pattern to MFN-2 in M, Hyb, T, and Hyb-NPs. Hyb-NPs had both characteristic proteins from T and M, implying that Hyb-NPs successfully inherited biological functions of both membranes. TPP-LND, targeting mitochondria to exert antitumor effects, was synthesized according to our previous study (*16*), and the structure was verified by ^1^H nuclear magnetic resonance (NMR) and mass spectrometry (figs. S1 to S3). Then, as shown in Fig. 2E, PEG, PLGA, and lecithin were used to prepare TPP-LND-loaded PEG-PLGA cores, and the cores were further coated with (i) R8 modified 1,2-distearoyl-*sn*-glycero-3-phosphoethanolamine-*N*-methoxy(polyethylene glycol) (DSPE-PEG-R8) and methoxy-modified 1,2-distearoyl-*sn*-glycero-3-phosphoethanolamine-*N*-methoxy(polyethylene glycol) (DSPE-PEG-OME) decorated hybrid membrane (TL/RH-NPs), (ii) DSPE-PEG-R8 and DSPE-PEG-OME decorated tumor cell membrane (TL/RT-NPs), and (iii) DSPE-PEG-R8 and DSPE-PEG-OME (TL/RP-NPs). After being coated with RH membrane, the nanoparticle size increased from 105.6 ± 1.68 nm for the PEG-PLGA core to 141.0 ± 5.05 nm for TL/RH-NPs (mitochondria-damaging nanoparticles). The zeta potential of TL/RH-NPs was −16.2 ± 0.79 mV, similar to that of the RH membrane, indicating a successful coating. In addition, TL/RH-NPs had a polydispersity Index (PDI) of 0.18 with a drug loading rate of 4.0% (w/w). Transmission electron microscopy (TEM) revealed the morphology of the prepared TL/RP-NPs and TL/RH-NPs. As shown in Fig. 2 (F and G), the nanoparticles appeared as uniformly dispersed spheres with distinct core-shell structures. Notably, TL/RH-NPs exhibited a thicker shell-like structure with visible bilayer membrane, further confirming the successful modification of mitochondria-targeting RH membrane on PEG-PLGA cores. As for the stability of nanoparticles (Fig. 2H), the particle sizes only experienced a slight change within 24 hours either in phosphate-buffered saline (PBS) or serum. The drug release of nanoparticles in Fig. 2I showed that TL/RH-NPs experienced a sustained release of TPP-LND to 80% within 48 hours. In contrast, TL/RP-NPs demonstrated faster drug release compared to TL/RH-NPs due to the absence of membrane decoration. Methylthiazolyldiphenyl-tetrazolium bromide (MTT) assay (Fig. 2J) showed that TL/RH-NPs had significantly stronger cytotoxicity compared to others, which might be attributed to the fact that the mitochondria-targeted drug delivery system precisely transported LND to its targets on the IMM, thereby potentiating its tumor killing ability. Further investigation on the mitochondria-targeting ability of TL/RH-NPs in Fig. 2 (K to M) illustrated that the whole mitochondria uptake of TL/RH-NPs outweighed TL/RP-NPs and TL/RT-NPs. Further quantitative analysis of drug distribution in the IMM and matrix demonstrated that TL/RH-NPs significantly enhanced drug delivery to these compartments compared to other nanoparticles, which might be attributed to the fact that the mitochondrial membrane of TL/RH-NPs fused with the outer mitochondrial membrane of the mitochondria, facilitating the interactions of TPP-LND with the IMM. This also explained the strongest cytotoxicity of TL/RH-NPs among all formulations in MTT assay.

### TL/RH-NPs had less efficacy in inhibiting metastasis with tumors characterized by higher level of mitocytosis

**Fig. 3.**
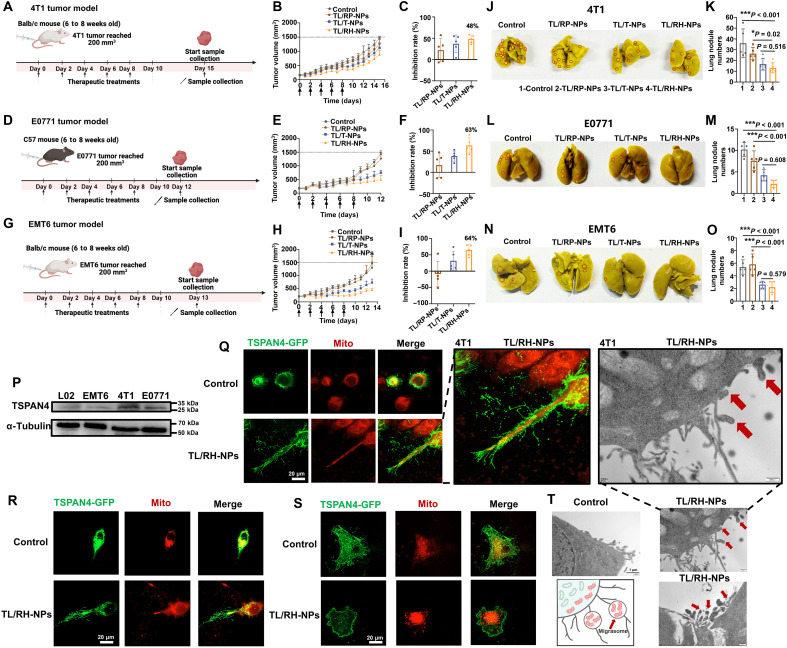
Antitumor efficiency of TL/RH-NPs against different breast tumor models and activation of compensatory mitocytosis. (**A**) To establish orthotopic 4T1 tumor models, Balb/c mice were inoculated with 4T1 cells. The tumor-bearing mice were then randomly assigned to four treatment groups: saline, TL/RP-NPs, TL/T-NPs, and TL/RH-NPs. The treatments were administered intravenously every other day for a total of five injections. Created in BioRender. Mahaha, M. (2026) https://BioRender.com/s1en2tw. (**B** and **C**) Tumor growth curve (B) and tumor inhibition rate (C) of 4T1 tumor-bearing Balb/c mice. (**D**) To establish orthotopic E0771 tumor models, C57 mice were inoculated with E0771 cells. Created in BioRender. Mahaha, M. (2026) https://BioRender.com/s1en2tw. (**E** and **F**) Tumor growth curve (E) and tumor inhibition rate (F) of E0771 tumor-bearing C57 mice. (**G**) To establish orthotopic EMT6 tumor models, Balb/c mice were inoculated with EMT6 cells. Created in BioRender. Mahaha, M. (2026) https://BioRender.com/s1en2tw. (**H** and **I**) Tumor growth curve (H) and tumor inhibition rate (I) of EMT6 tumor-bearing Balb/c mice. (**J**) Representative images of the excised lungs of 4T1 tumor–bearing Balb/c mice and (**K**) statistical analysis of tumor metastatic lung nodules. (**L**) Representative images of the excised lungs of E0771 tumor–bearing C57 mice and (**M**) statistical analysis of tumor metastatic lung nodules. (**N**) Representative images of the excised lungs of EMT6 tumor–bearing Balb/c mice and (**O**) statistical analysis of tumor metastatic lung nodules. (**P**) WB analysis of migrasome characteristic protein TSPAN4 in 4T1 cells, E0771 cells, EMT6 cells, and L02 cells. (**Q** to **S**) Laser confocal images of mitocytosis activation [cells are transfected with migrasome characteristic protein TSPAN4 (green), and mitochondria are marked by MitoTracker Red] after treatment with TL/RH-NPs in 4T1 cells (Q), E0771 cells (R), and EMT6 cells (S). (**T**) Bio-TEM images of TL/RH-NPs inducing mitocytosis in 4T1 cells. All results were presented as means ± SD, *n* = 5.

To investigate antitumor efficiency of TL/RH-NPs in vivo, orthotopic 4T1 and EMT6 breast tumor models were established in Balb/c mice, and orthotopic E0771 tumor model was established in C57 mice. As illustrated in Fig. 3 (A, D, and G), treatment was initiated when tumors reached 200 mm^3^ (mid-late stage), and the dosing regimen consisted of administration every other day for a total of five doses. As shown in Fig. 3 (A to I), in the same tumor model, tumor growth curves revealed that TL/RH-NPs had superior antitumor effects over TL/T-NPs and TL/RP-NPs, which proved the therapeutic advantages in delivering TPP-LND to the IMM. Further comparison across the three tumor models showed that TL/RH-NPs achieved good therapeutic outcomes in E0771 and EMT6 tumor models, with tumor inhibition rates reaching 63 and 64%, respectively. However, TL/RH-NPs exhibited relatively poor antitumor efficacy in 4T1 tumor model with an inhibition rate of 48%, exhibiting a tumor rebound after treatment. Similarly, tumor metastatic lung nodules in Fig. 3 (J to O) showed that the TL/RH-NP group had the fewest pulmonary nodules compared to other nanoparticles, demonstrating the most effective inhibition of tumor metastasis. When comparing the antimetastatic efficacy of TL/RH-NPs across the three different tumor models, they reduced the numbers of tumor metastatic lung nodules to just two in both E0771 and EMT6 models, indicating the significant suppression of tumor metastasis. In contrast, the 4T1 tumor model still had the numbers of tumor metastatic lung nodules above 13 after TL/RH-NP treatment. In summary, TL/RH-NPs exhibited relatively poor therapeutic performance in 4T1 tumor model compared to E0771 and EMT6 tumor models, particularly showing inferior antimetastatic effects.

To explore the reason for the differences in therapeutic efficiency observed across the three breast tumor models, we initially examined tetraspanin 4 (TSPAN4) expression. This protein regulates cell migration, and its localized enrichment at retraction fibers triggers migrasome formation (*17*), serving as a biomarker of migrasome. TSPAN4 was first assessed by WB assay, and normal L02 cells were introduced as negative control. As shown in Fig. 3P, migrasome expression was highest in 4T1 cells, followed by E0771, EMT6, and L02 cells, indicating the highest possibility for mitocytosis activation in 4T1 cells. Further investigation was conducted by confocal microscopy imaging, and 4T1, E0771, and EMT6 cells were transfected with TSPAN4–green fluorescent protein (GFP) and were concurrently stained with MitoTracker in red fluorescence. As shown in Fig. 3Q, after treatment with TL/RH-NPs, 4T1 cells exhibited significantly increased green fluorescence of TSPAN4, with abundant retraction fibers and migrasome visible. In addition, an obvious tendency of mitochondrial migration (red fluorescence) toward the retraction fibers was observed. More evidence was provided by marking the migrasomes and retraction fibers with wheat germ agglutinin (WGA) (fig. S4A). Confocal images revealed that TL/RH-NPs induced the formation of retraction fibers in 4T1 cells, with distinct vesicular structures forming at their tips. Mitochondria migrated from the cell periphery toward the fiber tips and accumulated within these vesicular structures. In contrast, the combination (Combo) effectively suppressed retraction fiber formation and trapped mitochondria within the cell body. Further investigation (fig. S4B) revealed that in the TL/RH-NP group, the vesicular structures at retraction fiber tips exhibited intense TSPAN4-GFP fluorescence (green), with clear mitochondrial fluorescence signals accumulated inside. Notably, neither the control group nor the Combo group exhibited this migrasome formation pattern. Collectively, these results clearly demonstrated obvious colocalization of mitochondria with migrasomes, which further proved the occurrence of mitocytosis. In Fig. 3R, E0771 cells treated with TL/RH-NPs also displayed prominent green fluorescence from TSPAN4, along with visible retraction fibers. Although mitochondria showed a tendency to migrate toward the retraction fibers, this directional movement was significantly attenuated compared to that in 4T1 cells. In Fig. 3S, EMT6 cells treated with TL/RH-NPs showed no increase in green fluorescence, no obvious retraction fibers, and only a slight tendency for mitochondria to migrate toward the cell membrane. These results indicated that among the three tumor models, 4T1 cells with the highest migrasome expression exhibited the most significant increase in the formation of retraction fibers and migrasomes after treatment with TL/RH-NPs, along with the most significant migrasome-mediated mitocytosis process. Bio-TEM was further used to observe mitocytosis activation in 4T1 cells after TL/RH-NP treatment. As shown in Fig. 3T, the control group exhibited several migrasome vesicle. In contrast, after stimulation by TL/RH-NPs, the formation of migrasome vesicles in 4T1 cells was significantly increased. Notably, these vesicles contained damaged mitochondria, confirming the occurrence of mitocytosis. This suggested that the high migrasome expression in 4T1 cells might contribute to the elevated mitocytosis activation, and the differences in mitocytosis activation among three tumor models lead to therapeutic differences. In the following study, we aimed to improve the therapeutic efficiency of mitochondria-targeted TL/RH-NPs by inhibiting compensatory mitocytosis in 4T1 tumor model.

### Characterization and targeting ability of migrasome-inhibiting nanoparticles

**Fig. 4.**
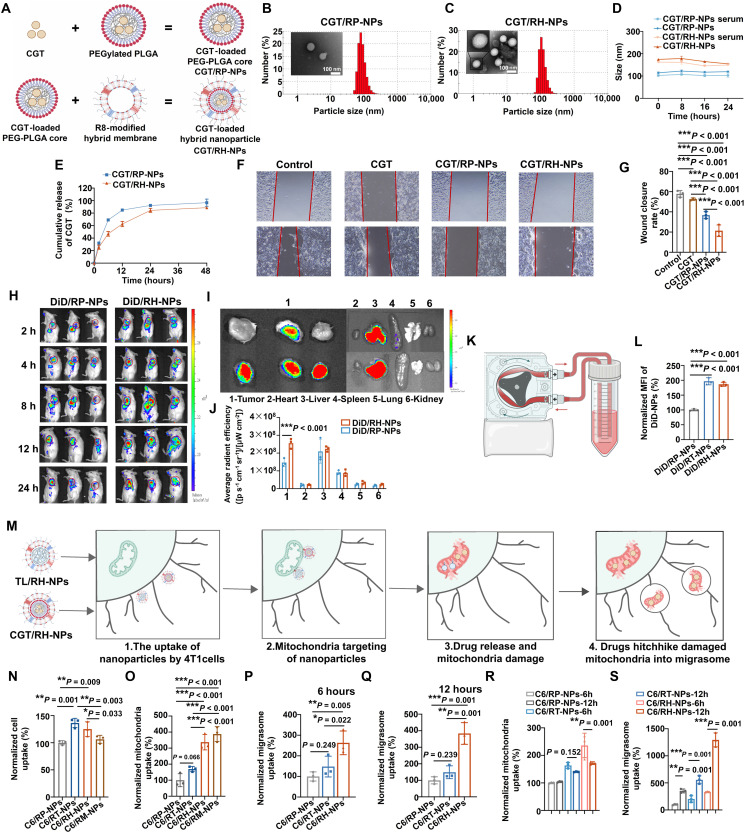
Characterization and targeting ability of migrasome-inhibiting nanoparticles. (**A**) Illustration of the preparation of CGT/RH-NPs. Created in BioRender. Mahaha, M. (2026) https://BioRender.com/s1en2tw. (**B** and **C**) Particle size distribution and TEM images of CGT/RP-NPs (B) and CGT/RH-NPs (C). (**D**) Stability of CGT/RP-NPs and CGT/RH-NPs at 37°C within 24 hours in PBS or 10% serum. (**E**) Cumulative release of CGT from CGT/RP-NPs or CGT/RH-NPs at 37°C within 24 hours in PBS. (**F**) Representative microscopy images of 4T1 cells treated with CGT, CGT/RP-NPs, or CGT/RH-NPs for 24 hours in wound healing assay and (**G**) statistical analysis of the wound healing rate. (**H**) Real-time fluorescence imaging of 4T1 tumor–bearing Balb/c mice after intravenous injection of DiD/RP-NPs or DiD/RH-NPs at 2, 4, 8, 12, and 24 hours. (**I**) Ex vivo fluorescence images of dissected tumors and organs at 24 hours postinjection. (**J**) Quantitative analysis of fluorescence signal for dissected tissues at 24 hours postinjection. (**K**) Schematic illustration of in vitro device for CTCs targeting investigation. 4T1 cells, DiD/RP-NPs, DiD/RT-NPs, or DiD/RH-NPs were mixed and cycled for 2 hours. Created in BioRender. Mahaha, M. (2026) https://BioRender.com/s1en2tw. (**L**) Statistical analysis of CTC uptake of DiD/RP-NPs, DiD/RT-NPs, or DiD/RH-NPs. (**M**) Illustration of RH-NPs delivering drugs into mitochondria and releasing drugs to hitchhike into migrasomes. (**N** and **O**) Normalized cell uptake (N) and mitochondria uptake (O) of C6/RP-NPs, C6/RT-NPs, C6/RH-NPs, or C6/RM-NPs in 4T1 cells at 6 hours. (**P** and **Q**) Normalized migrasome uptake of C6/RP-NPs, C6/RT-NPs, or C6/RH-NPs at 6 hours (P) or 12 hours (Q). (**R** and **S**) Normalized mitochondria uptake (R) and migrasome uptake (S) of C6/RP-NPs, C6/RT-NPs, or C6/RH-NPs at 6 hours and 12 hours. The mitochondria uptake of C6/RP-NPs at 6 hours is normalized as 100%. All results were presented as means ± SD, *n* = 3. h, hours. MFI, mean fluorescence intensity.

Migrasomes are enriched in integrins (*14*, *15*, *17*). Therefore, as illustrated in Fig. 4A, migrasome inhibiting nanoparticles were constructed by first loading integrin inhibitor CGT into PEG-PLGA core and then coating the core with mitochondria-targeting RH membrane (CGT/RH-NPs). CGT/RH-NPs had the particle size of 154.1 ± 4.60 nm and the zeta potential of −16.3 ± 0.89 mV. The PDI of CGT/RH-NPs was 0.27 with the drug loading rate of 5.3%. TEM was used to observe the morphology of CGT/RP-NPs and CGT/RH-NPs, as shown in Fig. 4 (B and C), and both nanoparticles exhibited uniformly dispersed spherical shapes with distinct core-shell structures. Compared to CGT/RP-NPs, CGT/RH-NPs displayed a thicker shell-like structure, and a clearer double-layer membrane structure was visible, further confirming successful membrane coating of RH. As for the stability of nanoparticles (Fig. 4D), the particle sizes only experienced a slight change within 24 hours either in PBS or serum. The drug release of nanoparticles in Fig. 4E showed that CGT/RH-NPs continuously released the CGT up to 80% within 24 hours. In contrast, CGT/RP-NPs demonstrated faster drug release compared to CGT/RH-NPs due to the absence of membrane coating. Wound healing assay was used to investigate the antimetastatic ability of CGT/RH-NPs. As shown in Fig. 4 (F and G), comparing to free CGT and CGT/RP-NPs without the decoration of membrane, CGT/RH-NPs with mitochondria-targeting capability most significantly suppressed the migration of 4T1 tumor cells. Subsequently, we systematically evaluated the targeting ability of RH-NPs. First, in vivo live imaging system was used to investigate the distribution of nanoparticles. As illustrated in Fig. 4 (H to J), the fluorescence of RP-NPs at tumor site declined by 24 hours. In contrast, RH-NPs demonstrated rapid tumor accumulation within 2 hours postinjection, peaking at 12 hours, with sustained fluorescence observed up to 24 hours. Notably, the fluorescence intensity of RH-NPs was obviously higher than that of RP-NPs at all time points, demonstrating the enhanced tumor-targeting efficiency and prolonged accumulation of RH-NPs than RP-NPs. Furthermore, a blood circulation mimetic device (Fig. 4K) was constructed and powered by a peristaltic pump to investigate the ability of nanoparticles to target circulating tumor cells (CTCs). 4T1 cells and 1,1-dioctadecyl-3,3,3,3-tetramethylindodicarbocyanine (DiD)–loaded NPs were mixed and cycled for 2 hours. As shown in Fig. 4L, flow cytometry results showed that the CTC uptake of RT-NPs and RH-NPs were 1.96- and 1.86-fold higher than that of RP-NPs, respectively, with no significant difference between the two. This demonstrated that RH endowed nanoparticles with the ability to target tumor cells migrated to the systemic circulation, implying their potential to inhibit hematogenous metastasis of tumor. Next, as shown in Fig. 4M, we further investigated the entire targeting process of RH-NPs, from cellular uptake to mitochondria-targeted drug delivery, and, last, the hitchhiking transport of drugs into migrasomes. The cell uptake of C6-labeled NPs was shown in Fig. 4N, and the cell uptake of RT-NPs and RH-NPs were significantly higher compared to RP-NPs and mitochondria membrane–coated nanoparticles (RM-NPs), demonstrating that nanoparticles with tumor cell membrane on the surface had active tumor-targeting capabilities. In Fig. 4O, RH-NPs exhibited 3.37- and 1.99-fold higher mitochondria uptake than RP-NPs and RT-NPs, with no significant difference in mitochondria uptake with RM-NPs, verifying their dual targeting abilities. The confocal microscopy images in fig. S5 also revealed that the colocalization index of RH-NPs with mitochondria was the highest, further verifying the mitochondria-targeting ability of RH-NPs. These experiments proved that RH-NPs have both tumor-targeting and mitochondria-targeting capabilities. During mitocytosis, damaged mitochondria are continuously transported into migrasomes. We therefore hypothesized that mitochondria-targeted nanoparticles, upon inducing mitochondrial damage, may likewise be incorporated into migrasomes through this process. Thus, the subcellular distribution of nanoparticles in mitochondria and migrasomes was investigated over time, and WB assay was conducted to prove the successful extraction of migrasomes (fig. S6). At 6 and 12 hours, the distribution of RH-NPs in migrasomes and mitochondria (Fig. 4, P and Q) was significantly higher than that of RP-NPs and RT-NPs, demonstrating its superior mitochondria targeting and migrasome targeting capabilities. Temporal analysis of organelle-specific distribution (Fig. 4, R and S) demonstrated a dynamic translocation of RH-NPs: Mitochondrial accumulation decreased, whereas migrasome accumulation increased 3.8-fold from 6 to 12 hours posttreatment, which might be attributed to the fact that TL/RH-NPs triggered migrasome biogenesis, facilitating the trafficking of damaged mitochondria containing RH-NPs into nascent migrasomes. Collectively, these findings demonstrated that RH-NPs not only exhibited dual-targeting capabilities to effectively accumulate in tumor cell and tumor mitochondria, but also showed enhanced migrasome accumulation following TL/RH-NP–induced mitocytosis activation, thereby emerging as an optimal nanoplatform for migrasome-targeted therapeutic intervention.

### Inhibiting metastasis through coordinated mitochondrial damage and mitocytosis blockade

**Fig. 5.**
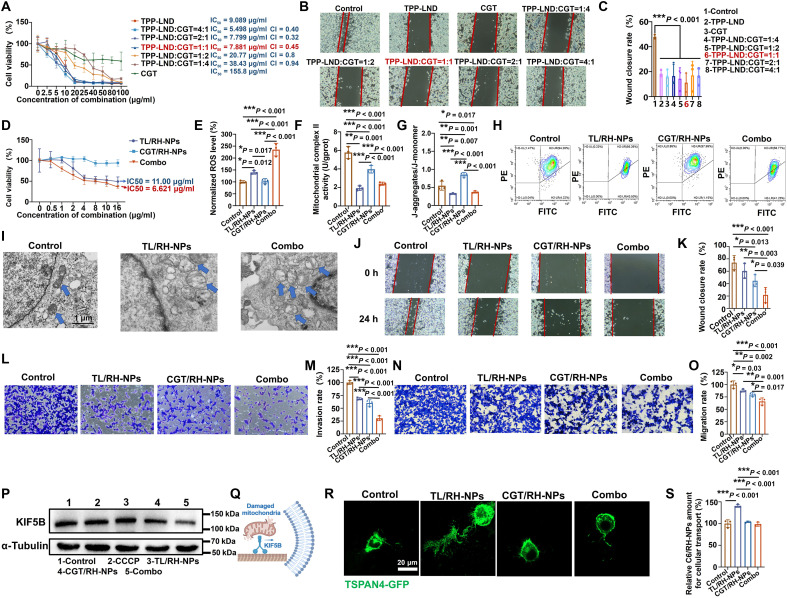
In vitro antitumor efficiency through coordinated mitochondrial damage and mitocytosis blockade. (**A**) 4T1 cell viability after treatment with different ratios of TPP-LND:CGT for 24 hours. (**B** and **C**) Representative microscopy images in wound healing assay and statistical analysis of the wound healing rate of 4T1 cells treated with different ratios of TPP-LND:CGT for 24 hours. (**D**) 4T1 cell viability after treatment with TL/RH-NPs, CGT/RH-NPs or Combo for 24 hours. (**E**) ROS generation of 4T1 cells after treatment with TL/RH-NPs, CGT/RH-NPs, or Combo. (**F**) Mitochondrial complex II activity of 4T1 cells treated with TL/RH-NPs, CGT/RH-NPs, or Combo. (**G**) Statistical analysis of J-aggregates/J-monomer of 4T1 cells and (**H**) flow cytometry images of J-aggregates (PE)/J-monomer (FITC) after treatment with TL/RH-NPs, CGT/RH-NPs, or Combo. (**I**) Bio-TEM images of TL/RH-NPs or Combo inducing mitochondrial damage in 4T1 cells. (**J**) Representative microscopy images in wound healing assay and (**K**) statistical analysis of the wound healing rate of 4T1 cells treated with TL/RH-NPs, CGT/RH-NPs, or Combo for 24 hours. (**L**) Representative microscopy images of the invaded 4T1 cells and (**M**) statistical analysis of the invasion rate of 4T1 cells treated with TL/RH-NPs, CGT/RH-NPs, or Combo for 24 hours. (**N**) Representative microscopy images of the migrated 4T1 cells and (**O**) statistical analysis of the migration rate of 4T1 cells treated with TL/RH-NPs, CGT/RH-NPs, or Combo for 24 hours. (**P**) WB analysis of KIF5B expression of 4T1 cell treated with CCCP, TL/RH-NPs, CGT/RH-NPs, or Combo. (**Q**) Illustration of KIF5B dragging damaged mitochondria to cell membrane. Created in BioRender. Mahaha, M. (2026) https://BioRender.com/s1en2tw. (**R**) Confocal images of 4T1 cells transfected with migrasome characteristic protein TSPAN4 after treatment with TL/RH-NPs, CGT/RH-NPs, or Combo. (**S**) Intracellular transport of damaged mitochondria between 4T1 cells after treatment with TL/RH-NPs, CGT/RH-NPs, or Combo. All results were presented as means ± SD, *n* = 3.

CGT/RH-NPs and TL/RH-NPs were combined to form a synergistic therapeutic regimen (designated Combo) for inhibition of tumor metastasis. First, MTT assays were used to screen the optimal combination ratio of the two drugs. As shown in Fig. 5A, when the ratios of CGT:TPP-LND were 1:4, 1:2, and 1:1, the combination therapies exhibited potent cytotoxicity with the combination index (CI) below 0.5 at these ratios, indicating synergistic effects between the two drugs. Then, the optimal drug combination ratio was further screened using the wound healing assay. As shown in Fig. 5 (B and C), the combination ratio of 1:1 exhibited the lowest wound closure rate, indicating superior antimetastatic efficacy. Collectively, the 1:1 ratio was ultimately selected as the optimal combination ratio for subsequent studies. The cytotoxicity of Combo was conducted by MTT assay, as illustrated in Fig. 5D, CGT/RH-NPs showed no cytotoxicity within the drug concentration of 0 to 16 μg/ml, and, in contrast, TL/RH-NPs and Combo showed significant cytotoxicity against 4T1 cells, suggesting that the cytotoxicity of Combo primarily originated from TL/RH-NPs. Moreover, the median inhibitory concentration (IC_50_) of Combo was lower than that of TL/RH-NPs, suggesting that CGT/RH-NPs synergistically enhanced the tumor-killing effects of TL/RH-NPs. Because moderate mitochondrial stress leads to mitocytosis, we chose the equivalent dose of TPP-LND (3 μg/ml) for TL/RH-NPs (the dose inducing mitocytosis in Fig. 3 without activating mitophagy as shown in fig. S7) for further study. To further investigate the antitumor mechanisms of Combo, flow cytometry assay was used to assess the ROS generation. The results in Fig. 5E showed that Combo most significantly increased the ROS level in 4T1 cells, which was attributed to the positively charged TPP-LND released by TL/RH-NPs interacting with mitochondrial respiratory chain complex II located on the IMM (Fig. 5F). The generated ROS led to mitochondrial damage, manifested as a decrease in mitochondrial membrane potential (Fig. 5, G and H). Meanwhile, Bio-TEM was used to investigate posttreatment morphological changes of mitochondria. As illustrated in Fig. 5I, mitochondria in the control group exhibited well-preserved internal architecture. In contrast, TL/RH-NPs and Combo induced severe mitochondrial damage, characterized by marked swelling, structural disintegration, and substantial loss of mitochondria. Collectively, these results demonstrated that Combo exerted potent antitumor activity through induction of mitochondrial damage, further proving the mitochondria-targeting advantages of Combo and synergistic effects of mitochondrial damaging and mitocytosis blockage.

Subsequently, the antimetastatic potential of Combo was evaluated. Wound healing assay (Fig. 5, J and K) revealed that while both TL/RH-NPs and CGT/RH-NPs monotherapies demonstrated moderate antimetastatic effects, Combo markedly suppressed tumor migration with the wound healing rate of only 20%. In addition, in vitro antitumor migration and invasion assays demonstrated similar trends (Fig. 5, L to O). Compared with the control group, both TL/RH-NPs and CGT/RH-NPs significantly reduced migration and invasion rates, indicating their potent inhibitory effects on tumor cell migration. Notably, Combo further decreased tumor cell migration and invasion rates, suggesting synergistic antimetastatic effects of the combination strategy. In addition, as shown in fig. S8, the treatment of siRNA for TSPAN4 (si-TSPAN4) alone significantly reduced 4T1 cell migration compared to control, demonstrating the prometastatic role of migrasomes. When combined with TL/RH-NPs, migration rate was further decreased to 44%, which is 43% lower than TL/RH-NPs alone (87%; Fig. 5O). These results further demonstrated that inhibiting migrasome formation enhances the antimetastatic efficacy of mitochondria targeted therapy, thereby providing a mechanistic rationale for our combination strategy. Next, investigation on antimetastatic mechanisms of Combo was conducted. Under mitochondrial damage, kinesin family member 5B (KIF5B) drives mitochondria to move toward the cell periphery for subsequent mitocytosis (*18*). Thus, the expression of KIF5B was investigated to indicate mitocytosis activation (Fig. 5, P and Q). Meanwhile, carbonyl cyanide 3-chlorophenylhydrazone (CCCP; a mitochondria-damaging agent) (*3*) was included as a positive control to induce mitocytosis. The results revealed that both CCCP and TL/RH-NPs up-regulated KIF5B expression compared to control group, which may be attributed to their activation of mitocytosis. In contrast, both CGT/RH-NPs and Combo significantly down-regulated KIF5B expression in 4T1 tumor cells, indicating the effective suppression of mitochondria exocytosis and consequent termination of mitocytosis. It was reported that CGT (with high affinity to integrin α5β1) (*19*) binds to α5-enriched migrasomes and disrupts the interaction between integrin and extracellular matrix (ECM) (*14*), which is a critical anchoring mechanism required for migrasome formation. As shown in fig. S9, after pretreatment of TL/RH-NPs, the CGT/RH-NPs and Combo significantly decreased the percentage of integrin α5–expressing cells, which might be attributed to the fact that RH-NPs could deliver CGT into integrin α5–enriched migrasomes, leading to more effective interaction with integrin α5. Furthermore, we transfected cells with TSPAN4-GFP, and confocal images (Fig. 5R) showed that TL/RH-NPs induced formation of abundant retraction fibers and migrasome in 4T1 cells. However, both CGT/RH-NP and Combo groups showed significantly attenuated green fluorescence, with no observable retraction fibers or distinct migrasomes, demonstrating effective suppression of migrasome biogenesis and inhibition of migrasome-mediated mitocytosis. Following mitocytosis activation, damaged mitochondria undergo intracellular transport. To monitor this intercellular transfer, we used mitochondria-targeted C6/RH-NPs as a fluorescent probe. As illustrated in fig. S10, donor 4T1 cells were coincubated with C6/RH-NPs and TL/RH-NPs (mitochondrial damage inducer/mitocytosis activator). Then, C6-labeled damaged mitochondria entered migrasomes and were expelled into the medium. After that, expelled migrasomes (containing mitochondria) were collected, treated with CGT/RH-NPs, Combo, or blank medium and incubated with recipient 4T1 cells. At last, the recipient cell uptake of migrasomes (containing mitochondria labeled by C6/RH-NPs) was quantified to assess mitocytosis-mediated mitochondria transfer. In control group, donor cells treated with C6/RH-NPs alone and all the following steps were the same. The results were shown in Fig. 5S, and, compared to control, TL/RH-NPs resulted in significantly increased uptake of migrasomes (containing mitochondria marked by C6/RH-NPs) by recipient cells, indicating the activation of mitocytosis and thereby promoting migrasome-mediated intercellular mitochondria transfer. However, when recipient cells were treated with either CGT/RH-NPs or Combo after TL/RH-NP–activated mitocytosis in donor cells, their ability to internalize damaged mitochondria was markedly reduced, demonstrating effective suppression of intercellular mitochondrial transfer. This inhibitory effect might be attributed to the ability of CGT to interfere with migrasome-ECM interactions. Collectively, these experiments systematically evaluated the mitocytosis process through three aspects: (i) mitochondria exocytosis (KIF5B expression), (ii) migrasome formation (TSPAN4 expression), and (iii) intercellular transfer of damaged mitochondria. The results demonstrated that although TL/RH-NPs significantly promoted mitocytosis, the combination administration of CGT/RH-NPs effectively suppressed this process, exhibiting potent antimetastatic effects.

### In vivo antitumor and antimetastatic efficiency

**Fig. 6.**
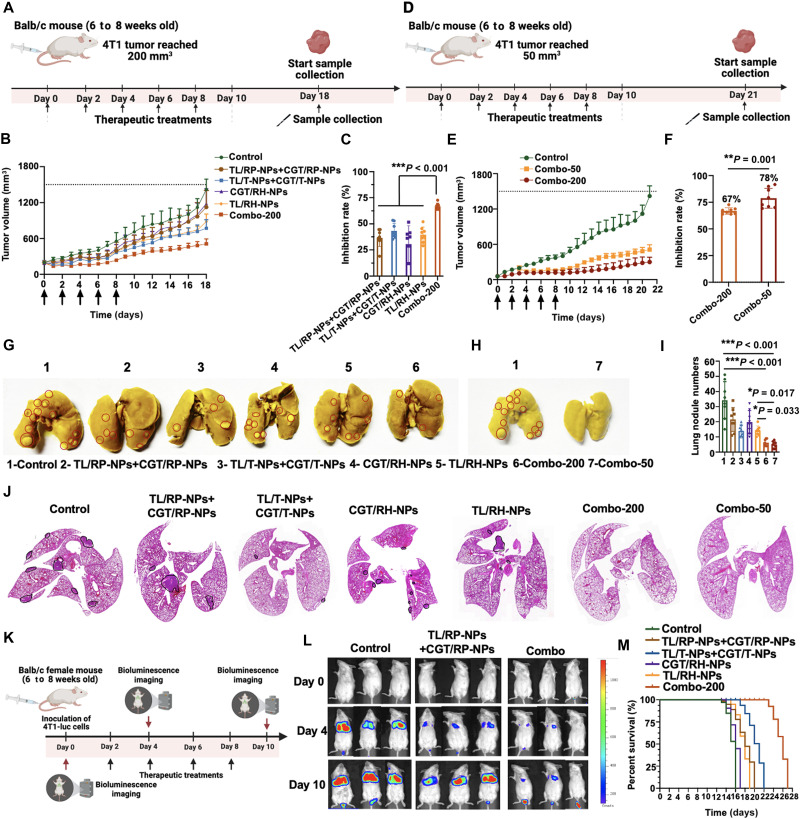
In vivo antitumor and antimetastatic efficiency of Combo. (**A**) To establish orthotopic 4T1 tumor models, Balb/c mice were inoculated with 4T1 cells. The tumor-bearing mice were then randomly assigned to four treatment groups: saline, TL/RP-NPs + CGT/RP-NPs, TL/T-NPs + CGT/T-NPs, CGT/RH-NPs, TL/RH-NPs, and Combo-200. The therapeutics were administered intravenously every other day for a total of five injections when tumor volume reached 200 mm^3^. Created in BioRender. Mahaha, M. (2026) https://BioRender.com/s1en2tw. (**B**) Tumor growth curve of 4T1 tumor–bearing Balb/c mice treated with saline, CGT/RH-NPs, TL/RP-NPs + CGT/RP-NPs, TL/T-NPs + CGT/T-NPs, TL/RH-NPs, or Combo-200 (*n* = 8). (**C**) Tumor inhibition rate of 4T1 tumor-bearing Balb/c mice treated with saline, TL/RP-NPs + CGT/RP-NPs, TL/T-NPs + CGT/T-NPs, TL/RH-NPs, CGT/RH-NPs, and Combo-200 (*n* = 8). (**D**) To establish orthotopic 4T1 tumor models, Balb/c mice were inoculated with 4T1 cells. The tumor-bearing mice were then randomly assigned and administered intravenously with Combo-50. The therapeutics were administered every other day for a total of five injections when tumor volume reached 50 mm^3^. Created in BioRender. Mahaha, M. (2026) https://BioRender.com/s1en2tw. (**E**) Tumor growth curve of 4T1 tumor–bearing Balb/c mice treated with saline, Combo-200, or Combo-50 (*n* = 8). (**F**) Tumor inhibition rate of 4T1 tumor–bearing Balb/c mice treated with saline, Combo-200, or Combo-50 (*n* = 8). (**G**) The representative lung images of saline, TL/RP-NPs + CGT/RP-NPs, TL/T-NPs + CGT/T-NPs, CGT/RH-NPs, TL/RH-NPs, and Combo-200. (**H**) The representative lung images of saline and Combo-50. (**I**) Statistical analysis of tumor metastatic lung nodes of 4T1 tumor–bearing Balb/c mice (*n* = 8). (**J**) H&E staining images of lungs for 4T1 tumor–bearing Balb/c mice. (**K**) The metastasis of tail vein–injected 4T1-Luc cells to the lungs was monitored by in vivo imaging. Created in BioRender. Mahaha, M. (2026) https://BioRender.com/s1en2tw. (**L**) Real-time bioluminescence imaging of lung metastasis for 4T1-Luc tumor cells in Balb/c mice on days 4 and 10 (*n* = 3). (**M**) The survival rates of 4T1 tumor–bearing Balb/c mice (*n* = 8). All results were presented as means ± SD.

Last, we investigated the in vivo antitumor and antimetastatic efficiency of nanoparticles. In this experiment, treatment regimen (Fig. 6A) was initiated when tumors reached 200 mm^3^ (mid-late stage), and the dosing regimen consisted of administration every other day for a total of five doses to evaluate the antimetastatic efficacy of the nanoparticles. As shown in Fig. 6, B and C, CGT/RH-NPs exhibited limited antitumor effects. Similarly, TL/RH-NPs displayed moderate antitumor effects (tumor inhibition rate of 39%) and notable posttreatment rebound. However, Combo (Combo-200) achieved optimal therapeutic outcomes, with the tumor inhibition rate of 67%. These results demonstrated that suppressing compensatory mitocytosis enhanced the antitumor efficacy of mitochondria-targeted drug delivery systems. Moreover, non–membrane-coated (CGT/RP-NPs + TL/RP-NPs) and tumor membrane–coated groups (CGT/T-NPs + TL/T-NPs) showed inferior antitumor activity, again suggesting the superiority of hybrid membrane coating. To further assess therapeutic potential of Combo, the treatment regimen (Fig. 6D) was initiated at early-stage tumors (50 mm^3^), and the dosing regimen also consisted of administration every other day for a total of five doses. As demonstrated in Fig. 6 (E and F), Combo-50 (early intervention) enhanced the tumor inhibition rate from 67% (achieved by Combo-200 at mid-late stage) to 78%, indicating superior antitumor efficacy of the early intervention strategy. Quantitative analysis of tumor metastatic lung nodules (Fig. 6, G to I) revealed that the control group developed metastatic nodules of 34, while TL/RH-NPs significantly reduced metastatic nodule numbers to 14. Notably, Combo-200 and Combo-50 demonstrated superior antimetastatic efficacy, suppressing metastatic nodule to merely 6 and 5, respectively, highlighting their capacity to inhibit primary tumor dissemination to lungs. Hematoxylin and eosin (H&E) staining of lung sections in Fig. 6J revealed that the control group exhibited the most obvious metastatic foci with the most metastatic nodule numbers. While CGT/RP-NPs + TL/RP-NPs, CGT/T-NPs + TL/T-NPs, CGT/RH-NPs, and TL/RH-NPs all showed detectable tumor metastatic foci, no visible tumor metastatic foci were observed in either the Combo-200 or Combo-50 group, demonstrating the superior antimetastatic efficacy of Combo.

To further evaluate tumor hematogenous migration, luciferase-expressed 4T1 (4T1-Luc) cells were intravenously injected into Balb/c mice, and drug administration was started on the second day (Fig. 6K) to validate the efficiency of Combo in suppressing hematogenous metastasis. As shown in Fig. 6L, on day 0 postinoculation with 4T1-Luc cells, no tumor-derived bioluminescence was detected in any groups. By day 4, control group exhibited strong bioluminescence localized primarily in lungs, indicating successful colonization of intravenously injected 4T1-Luc cells to form pulmonary metastasis. At this time point, both CGT/RP-NPs + TL/RP-NPs and Combo groups showed faint pulmonary bioluminescence, with significantly lower intensity in Combo. On day 10, the control group demonstrated markedly enhanced bioluminescence with extensive pulmonary signals, confirming massive metastatic colonization. The CGT/RP-NP + TL/RP-NP group also developed substantial metastasis. In comparison, Combo maintained only minimal signals in lungs. These results demonstrated that Combo not only suppressed primary tumor metastasis but also inhibited the colonization of CTCs in distant organs. This aligns with our in vitro findings that Combo effectively targets CTCs (Fig. 4L), thereby preventing their metastatic seeding in lungs. The goal of antitumor and antimetastatic therapy is to prolong the survival of tumor-bearing mice. Therefore, the survival outcomes following nanoparticle treatment were evaluated. As shown in Fig. 6M, control group exhibited a median survival of only 15 days. However, Combo significantly prolonged median survival to 24 days.

Last, the biosafety of the nanodelivery system was evaluated. As shown in fig. S11A, no significant changes in body weight were observed in mice throughout the treatment period. H&E staining images (fig. S11B) revealed no obvious morphological damage in major organs. Furthermore, serum biochemical analysis (fig. S11C) confirmed the absence of functional impairment in the heart, liver, or kidneys.

## DISCUSSION

During cell migration, tensile stress at the trailing edge ruptures retraction fibers, triggering plasma membrane budding at fiber ends to form 0.5- to 3-μm vesicles (*1*). These migration-dependent structures, termed migrasomes, exhibit three core functions (*3*, *20*): (i) Cellular homeostasis maintenance: Damaged mitochondria are expelled via migrasomes to preserve intracellular mitochondrial homeostasis and cell viability. (ii) Intercellular signaling: Migrasomes release chemokines, cytokines, and growth factors into the tumor microenvironment after exocytosis. (iii) Material transfer: Recipient cells engulf expelled migrasomes, enabling intercellular communication during migration. Crucially, in highly migratory cells under elevated mitochondrial stress (elevated energy demands, respiratory rates, and ROS production versus quiescent cells), mitocytosis emerges as a specialized migrasome-mediated adaptation (*3*). When mitochondria-targeted drugs disrupt the mitochondria, the impaired mitochondria enter migrasomes for exocytosis, sustaining homeostasis via this compensatory pathway. Unlike well-established mitophagy (*21*) (mitochondrial compensatory pathway that directly eliminates damaged mitochondria), migrasomes might function as signaling carriers that modulate the tumor microenvironment during mitocytosis.

In our study, we try to elucidate the unknown effects of mitocytosis modulation on the antimetastatic efficacy of mitochondria-targeted therapy in tumors. In WB experiments, the expression of migrasome characteristic protein TSPAN4 was as follows: highly migratory 4T1 cells > moderately migratory E0771 cells > weakly migratory EMT6 cells > normal L02 cells. This indicated that highly migratory 4T1 cells required more migrasomes to maintain homeostasis. Consequently, when stimulated by mitochondria-targeted drug delivery system (TL/RH-NPs), 4T1 cells exhibited significantly activated mitocytosis (validated by Bio-TEM and transfection assays). In contrast, the other two cell lines (E0771 and EMT6) showed lower compensatory mitocytosis activation posttreatment (transfection data), which was attributed to their low migrasome expression. The differential activation of migrasome-mediated compensatory mitocytosis directly affected the therapeutic efficacy of TL/RH-NPs across tumor models with the lowest tumor inhibition rate of 48% and the highest number of tumor metastatic lung nodule of 13 in 4T1 tumor model. In 4T1 tumors with high migrasome expression, mitocytosis activation might be a key factor limiting the antimetastatic efficacy of mitochondria-targeted therapy. In subsequent studies, we combined mitocytosis inhibition with mitochondrial damage, reduced in vitro invasion and migration rates of 4T1 cells to 20 to 30%, and decreased tumor metastatic lung nodules in 4T1 tumor model from 34 (control group) to 5 in vivo, demonstrating that suppressing the compensatory mitocytosis significantly potentiated the antimetastatic efficacy of mitochondria-targeted therapy.

While mitocytosis inhibition exhibits great therapeutic potential to enhance mitochondria-targeted antitumor metastatic therapy, developing effective regulatory strategies is a critical challenge in our initial system design. It was reported that migrasomes are enriched with integrins (*14*, *15*), prompting us to inhibit migrasome-mediated mitocytosis in highly migratory 4T1 cells using the integrin inhibitor like CGT. However, as integrins are ubiquitously expressed in tumors, simply delivering CGT to tumor sites might not specifically disrupt migrasome biogenesis for effective mitocytosis suppression. We therefore pursued migrasome-specific delivery to ensure therapeutic efficiency. Critically, the absence of direct migrasome-targeting strategies leads us to develop an indirect approach leveraging a key phenomenon: During mitocytosis, damaged mitochondria actively enter migrasomes. Upon mitocytosis activation by mitochondria-damaging TL/RH-NPs, a codelivered mitochondria-targeted system containing migrasome inhibitors is designed to hitchhike with damaged mitochondria into migrasomes. We thus encapsulate CGT into RH-NPs (CGT/RH-NPs) and hypothesize that during TL/RH-NP–triggered mitocytosis, CGT/RH-NPs cotransport with impaired mitochondria to suppress migrasome function. This hitchhiking strategy provides a previously unknown approach for migrasome-targeted therapeutic intervention.

However, the compensatory pathways mediated by migrasomes, and their underlying mechanisms require further investigation. The specific signaling pathways through which migrasomes promote tumor cell migration remain unknown, and elucidating their roles in metastasis may reveal previously unknown therapeutic targets for antimetastatic strategies. Moreover, current understanding of migrasome structure and function remains limited, significantly hindering the development and application of migrasome-targeted drug delivery systems. Designing delivery platforms to directly target and modulate migrasomes represents a promising antimetastatic approach. Further exploration of these areas could unlock therapeutic potential of migrasomes in disease treatment.

## MATERIALS AND METHODS

### Materials and reagents

Fetal bovine serum (FBS; catalog no. CE000-N031) was purchased from ExCell Biotechnology Co., Ltd. (Suzhou, China). 4′,6-Diamidino-2-phenylindole (DAPI; catalog no. C0060), PBS (catalog no. P1020), streptomycin and penicillin liquid (catalog no. P1400), and trypsin (catalog no. T8150) were purchased from Solarbio Technology Co., Ltd. (Beijing, China). RPMI 1640 (catalog no. L210KJ) medium was purchased from Yuanpei Biotechnology Co., Ltd. (Shanghai, China). PLGA (50:50; viscosity, 0.18 to 0.25 dl/g) with one methoxy end group (catalog no. DG-50DLG025) was purchased from Daigang BIO Engineer Limited Co., Ltd. (Jinan, China). R8-cys (customized) was purchased from Yuantai Biotechnology Co., Ltd., Nanjing, China. Soybean phospholipid (catalog no. MB5129-1) was purchased from Dalian Meilun Biotechnology Co., Ltd. (Shanghai, China). DSPE-PEG2000-methoxyl (catalog no. PS1-E1-2K) and DSPE-PEG2000-maleimide (catalog no. PS2-MDE-1K) were purchased from Ponsure Biotechnology, China. CGT (catalog no. BD628662) was purchased from Bide Pharmaceutical Company (Shanghai, China). LND (catalog no. L129342), 1-(3-dimethylaminopropyl)-3-ethylcarbodiimide hydrochloride (catalog no. D466469), 3-bromopropylamine (catalog no. 5003-71-4), and triphenylphosphine (catalog no. 603-35-0) were purchased from Aladdin Biochemical Technology Co., Ltd. (Shanghai, China). Protease inhibitor cocktail (catalog no. K1010) was purchased from Apexbio, USA. Bicinchoninic acid protein assay kit (catalog no. P0010), ROS assay kit (catalog no. S0033M), enhanced mitochondrial membrane potential assay kit (catalog no. kitC2003S), and mitochondria isolation kit (catalog no. C3601) were acquired from Beyotime Biotechnology Co., Ltd. (Shanghai, China). Mitochondrial respiration complex II assay kit (catalog no. E-BC-K835-M) was acquired from Elabscience Biotechnology Co., Ltd. (Wuhan, China). Coumarin-6 (catalog no. 44236) was purchased from Sigma-Aldrich (Germany), and DiD (catalog no. V22887) was purchased from Invitrogen (USA). MitoTracker Red (catalog no. 720833ES) and MitoTracker Deep Red (catalog no. 720833ES50) were purchased from Yeasen Biotechnology Co., Ltd. (Shanghai, China). WGA (catalog no. MP6325) was purchased from Maokang Biotechnology Co., Ltd. (Shanghai, China). Anti-α-tubulin (catalog no. HA721914), anti-Na-K-ATPase (catalog no. HA750194), anti-GM130 (catalog no. HA721282), and anti-Tsg101 (catalog no. ET1701-59) antibodies were purchased from Hua’an Biotechnology Co., Ltd. (Hangzhou, China). CD44 (catalog no. WL03531) antibody was purchased from Wanlei Biotechnology Co., Ltd. (Shenyang, China). Anti-MFN-2 (catalog no. A19678), anti-KIF5B (catalog no. A20928), anti-NDST1 (catalog no. A17498), and anti-TSPAN4 (catalog no. A10253) antibodies were purchased from ABclonal Biotechnology Co., Ltd. (Wuhan, China). Anti-LC-3B (catalog no. 83506) and anti-p62 (catalog no. 39749) antibodies were purchased from Cell Signaling Technology, Inc. (USA). Anti-integrin α5 [fluorescein isothiocyanate (FITC)] (catalog no. 11-0493-81) was purchased from Thermo Fisher Scientific (USA). Opti-MEM (catalog no. 31985062) and lipofectamine 3000 (catalog no. L3000015) were purchased from Thermo Fisher Scientific (USA). M5 Universal RNA Mini Kit (catalog no. MF036-01) were purchased from Mei5 Biotechnology, Co., Ltd. (Beijing, China).

*TSPAN4* in pEGFP-N1 plasmid (TSPAN4-GFP, species: mouse, *TSPAN4* sequence: 5′-ATGGCGCGCGCCTGCCTCCAGGCCGTCAAGTACCTCATGTTCGCCTTCAACCTGCTCTTCTGGCTGGGAGGCTGTGGCGTGCTGGGTGTCGGCATCTGGCTGGCCGCCACACAGGGGAGCTTCGCCACGCTGTCCTCTTCCTTCCCGTCCCTGTCGGCTGCCAACTTGCTCATCATCACCGGCGCCTTTGTCATGGCCATCGGCTTCGTGGGCTGCCTGGGTGCCATCAAGGAGAACAAGTGCCTCCTGCTCACTTTCTTCCTGCTGCTGCTGCTGGTGTTCCTGCTGGAGGCCACCATCGCCATCCTCTTCTTCGCCTACACGGACAAGATTGACAGGTATGCCCAGCAAGACCTGAAGAAAGGCTTGCACCTGTACGGCACGCAGGGCAACGTGGGCCTCACCAACGCCTGGAGCATCATCCAGACCGACTTCCGCTGCTGTGGCGTCTCCAACTACACTGACTGGTTCGAGGTGTACAACGCCACGCGGGTACCTGACTCCTGCTGCTTGGAGTTCAGTGAGAGCTGTGGGCTGCACGCCCCCGGCACCTGGTGGAAGGCGCCGTGCTACGAGACGGTGAAGGTGTGGCTTCAGGAGAACCTGCTGGCTGTGGGCATCTTTGGGCTGTGCACGGCGCTGGTGCAGATCCTGGGCCTGACCTTCGCCATGACCATGTACTGCCAAGTGGTCAAGGCAGACACCTACTGCGCGTAG-3′), *si-TSPAN4* (species: mouse, sense: 5′-CAGUGAUAGCUGUGGGUUATT-3′; antisense: 5′-UAACCCACAGCUAUCACUGTT-3′), negative control RNA (si-NC, sense: 5′-UUCUCCGAACGUGUCACGUTT-3′; antisense: 5′-ACGUGACACGUUCGGAGAATT-3′), the primer for *TSPAN4* (species: mouse, sequence: *TSPAN4-F*: 5′-AGGTGGTAAAGGCGGACA-3′; *TSPAN4-R*: 5′-ATGGATGCTGGGTCTCT-3′), and the primer for glyceraldehyde-3-phosphate dehydrogenase (*GAPDH*) (species: mouse, sequence: *GAPDH-F*: 5′-GGATGCTGCCCTTACCC-3′; *GAPDH-R*: 5′-GTTCACACCGACCTTCACC-3′) were synthesized by Sangon Biotechnology Co., Ltd., Shanghai, China.

### Cells and animals

4T1 murine breast cancer cells (catalog no. CL-0007) and EMT6 murine breast cancer cells (catalog no. CL-0573) were obtained from Pricella Life Science and Technology Co., Ltd. (Shanghai, China), E0771 murine breast cancer cells (catalog no. SNL-447) were obtained from Sunncell Biotechnology Co., Ltd. (Wuhan, China), and 4T1-Luc cells (catalog no. YC-B004-Luc-P) were obtained from Ubigene Biosciences Co., Ltd. (Guangzhou, China). 4T1, E0771, and EMT6 cells were grown in RPMI 1640 medium supplemented with 10% FBS and 1% antibiotics (penicillin and streptomycin). These cells were incubated at 37°C with 5% CO_2_ (v/v) in a thermostatic cell incubator.

Female Balb/c mice and C57 mice (specific pathogen free, 6 to 8 weeks old, 20 ± 2 g) were obtained from Sipeifu Biotechnology Co., Ltd., Beijing, China. Mice were housed under SPF conditions on a 12-hour light/12-hour dark cycle with food and water provided ad libitum. All of the animal experiments were approved by the Medical Ethics Committee of Sichuan University, and the animal experiments were conducted in the Animal Laboratory of West China School of Pharmacy in Sichuan University [accreditation number: SYXK (Chuan) 2018-113].

### Extraction and characterization of mitochondria membrane and cancer cell membrane

4T1 cancer cell membrane and mitochondrial membrane were prepared following our previously published protocols (*8*, *22*). Membrane vesicle size and zeta potential were measured by dynamic light scattering (Zeta Sizer Nano ZS90, Malvern, UK).

For SDS-PAGE analysis, purified 4T1 cell membrane (TM), mitochondrial membrane (MM), and hybrid membrane (TM:MM = 1:1) were separated by electrophoresis and visualized with Coomassie Brilliant Blue staining. For WB, membrane samples at varying ratios were solubilized in radioimmunoprecipitation assay (RIPA) buffer, heated, and separated on 10% polyacrylamide gels. Protein samples were transferred to polyvinylidene difluoride membranes, incubated with primary antibodies (anti-CD44, anti-MFN-2, and anti-Na-K-ATPase) and secondary antibodies, and then detected using a ChemDoc XRS system (Bio-Rad, USA).

### Synthesis and characterization of TPP-LND

The synthetic route of TPP-LND followed our previously published protocols and was shown in fig. S1 (*16*, *23*). First, BrCH_2_CH_2_NH_3_^+^Br^−^ (600 mg, 2.73 mmol) and TPP (500 mg, 1.91 mmol) were added to CH_3_CN (20 ml), heated at 80°C, stirred, and refluxed for 24 hours. Ten milliliters of *n*-hexane was added to precipitate the product, and then, the precipitate was washed with 10 ml of *n*-hexane to obtain an oily precipitate. The oily precipitate was dissolved in isopropanol, and then, the product was precipitated with anhydrous ether and further recrystallized to obtain TPP-NH_2_ (550 mg, 1.375 mol). Subsequently, TPP-NH2 (75 mg, 0.186 mmol), LND (50 mg, 0.155 mmol), 1-Hydroxybenzotriazole (18.8 mg), 1-Ethyl-3-(3-dimethylaminopropyl)carbodiimide (59.5 mg), and *N*,*N*-Diisopropylethylamine (80 μl) were dissolved in a mixed solvent of dichloromethane and methanol. The reaction was stirred at room temperature for 48 hours, and the product TPP-LND was purified by silica gel column (eluent ethyl acetate:methanol = 20:1). The purified product was dried in vacuum, and Bruker NMR spectrometer was used to record ^1^H-NMR spectra. In addition, electrospray ionization mass spectrometry was performed.

### Preparation and characterization of nanoparticles

First, DSPE-PEG-MAL and R8-Cys were subjected to a maleimide-thiol coupling reaction in *N*,*N*′-dimethylformamide at a mass ratio of 2:1 for 24 hours according to previous protocol in our laboratory (*24*, *25*) and were dialyzed using a dialysis bag with a molecular weight of 1000 kDa. After 24 hours, the solution in the dialysis bag was collected and freeze-dried to obtain white DSPE-PEG-R8 powder. Then DSPE-PEG-R8 and DSPE-PEG-OME were decorated on membranes through previously reported extrusion approach.

The PEG-PLGA cores were obtained through nanoprecipitation method: Briefly, 2.0 mg of PLGA, 0.4 mg of DSPE-PEG2000-OME, and drugs (150 μg of TPP-LND) or 2.0 mg of PLGA, 1.2 mg of DSPE-PEG2000-OME, and drugs [500 μg of CGT, 6.67 μg of DiD, or 6.67 μg of C6 dissolved in dimethyl sulfoxide (DMSO)] were mixed with 0.4 mg of lecithin dissolved in methyl alcohol as the oil phase. The oil phase was then added dropwise into stirring deionized water to allow nanoprecipitation. The resulting nanoparticle suspension was concentrated via ultrafiltration using an ultrafiltration tube with a 30-kDa molecular weight. As a control particle, the R8-modified PEG-PLGA NPs were also prepared through a nanoprecipitation method. In detail, 2.0 mg of PLGA, 0.2 mg of DSPE-PEG-R8, 0.6 mg of DSPE-PEG-OME, and 0.4 mg of lecithin dissolved in methyl alcohol were mixed as the oil phase. Then, the DMSO solution was added dropwise into 2.0 ml of deionized water for 10 min at room temperature to obtain RP-NPs.

Membrane coating was achieved by combining vesicles with PEG-PLGA cores (polymer-to-protein mass ratio = 2:1) and extruding the mixture through 200-nm polycarbonate membranes. Tumor cell membrane, R8-modified tumor cell membrane, and R8-modified hybrid membrane were prepared using T-NPs, RT-NPs, and RH-NPs.

The hydrodynamic diameter and zeta potential were determined by dynamic light scattering using a Zeta Sizer Nano ZS90 (Malvern, UK). Nanoparticle morphology was observed under TEM (model H-600, Hitachi, Japan). TPP-LND loading capacity and encapsulation efficiency of the nanoparticles were assessed by high-performance liquid chromatography (HPLC) using a Diamonsil C18 column (150 mm by 4.6 mm, 5 μm). The mobile phase was a 60:40 (v/v) mixture of acetonitrile:water containing 0.1% trifluoroacetic acid. TPP-LND was detected at 300 nm. CGT loading capacity and encapsulation efficiency of the nanoparticles were assessed by HPLC using a Diamonsil C18 column (150 mm by 4.6 mm, 5 μm). The mobile phase followed the gradient elution program in Table 1. CGT was detected at 220 nm.

**Table 1.** Gradient elution program for CGT.

Time (min)	Mobile phase A: 0.1% trifluoroacetic acid (TFA) in water (%)	Mobile phase B: 0.1% TFA in acetonitrile (%)
**0**	90	10
**13.5**	52	48
**14.5**	90	10
**20**	90	10

The drug loading efficiency (DL %) was calculated according to the following formula: DL % = (amount of drug loaded in nanoparticles)/nanoparticles weight) × 100%. For in vitro stability evaluation, TL/RP-NPs, TL/RH-NPs, CGT/RP-NPs, and CGT/RH-NPs were dispersed in PBS (pH 7.4), and the particle size of nanoparticles was measured at different time points. For in vitro drug release experiment, TL/RP-NPs, TL/RH-NPs, CGT/RP-NPs, and CGT/RH-NPs were dispersed in PBS (pH 7.4) in the dialysis tube (molecular weight cutoff of 10 kDa), and the drug was quantified by HPLC.

### In vitro cytotoxicity of nanoparticles

For evaluating the cytotoxicity of TPP-LND, nanoparticles, and the combination of CGT and TPP-LND against 4T1 cells, MTT method was applied. 4T1 cells were seeded onto 96-well plates. After 24 hours of incubation, series concentrations of drugs were added into the corresponding well. After 24 hours treatment, MTT solution was added into each well and incubated for another 4 hours before 150 μl of DMSO was added and the absorbance at 570 nm was measured via a microplate reader (Varioskan LUX, Thermo Fisher Scientific). To evaluate the synergistic effect of CGT and TPP-LND, the CI based on the synergistic level was calculated by CompuSyn software. The classifications of synergy are synergistic (CI < 1), additive (CI = 1), or antagonistic (CI > 1).

### In vitro targeting ability of nanoparticles

To investigate the ability of TL/RH-NPs to target IMM, 4T1 cells were then incubated with TL/RP-NPs, TL/RT-NPs, and TL/RH-NPs (equivalent concentration of TPP-LND: 6 μg/ml) for 4 hours. Mitochondria were isolated following the protocol in mitochondrial membrane extraction. Subsequently, mitochondrial fractions were treated with 1% digitonin solution for 10 min to disrupt the outer mitochondrial membrane according to our previous study, followed by centrifugation at 10,000*g* for 10 min. The supernatant containing mitochondrial intermembrane space and outer membrane components was subjected to drug extraction using 100 μl of methanol. Concurrently, the pellet containing IMM and mitochondrial matrix constituents was extracted with 100 μl of methanol. TPP-LND in both fractions was quantified via HPLC.

To assess nanoparticle biodistribution, DiD-labeled RP-NPs and RH-NPs were intravenously administered to 4T1 tumor–bearing mice (DiD dose: 125 μg/kg, *n* = 3) when tumors reached 500 mm^3^. Fluorescence imaging was performed using an IVIS Spectrum In Vivo Imaging System (PerkinElmer, Lumina 3, USA) at predetermined time points. At 24 hours postinjection, animals were euthanized, and tumors plus major organs were harvested for ex vivo fluorescence analysis under identical imaging parameters.

To investigate the ability of nanoparticles to target circulating cancer cells in vitro, a circulating pump was used to mimic the process of nanoparticles targeting circulating cancer cells. Specifically, 1 × 10^6^ 4T1 cells, DiD/RP-NPs, DiD/RT-NPs, and DiD/RH-NPs were mixed and circulated within the pump for 6 hours. After circulation, the mixture was concentrated (3000 rpm for 3 min) and washed with PBS. Subsequently, DiD fluorescence in 4T1 cells were assessed by flow cytometry.

To investigate the cellular and mitochondrial uptake of nanoparticles, 4T1 cells were first incubated with C6/RP-NPs, C6/RT-NPs, C6/RH-NPs, and C6/RM-NPs for 6 hours (equivalent C6 concentration of 2.5 μg/ml). Cells were harvested and analyzed by flow cytometry (Beckman CytoFLEX, CA, USA). As for the mitochondria targeting ability of nanoparticles, 4T1 cells were incubated with C6/RP-NPs, C6/RT-NPs, C6/RH-NPs, and C6/RM-NPs for 6 hours (equivalent C6 concentration of 2.5 μg/ml). Then, the mitochondria of 4T1 cells were extracted according to the abovementioned protocol for mitochondria extraction and immediately measured by flow cytometry. For confocal images, 4T1 cells were incubated with C6/RP-NPs, C6/RT-NPs, C6/RH-NPs, and C6/RM-NPs for 6 hours (equivalent C6 concentration 2.5 μg/ml). Then, cells were washed and stained with MitoTracker Red for 45 min. After that, cells were washed, fixed with 4% paraformaldehyde (PFA), and stained with DAPI (5 μg/ml) for 5 min. The fluorescence images were analyzed by confocal laser scanning microscopy (CLSM).

To evaluate cellular and mitochondrial uptake, 4T1 cells were treated with C6-labeled RP-NPs, RT-NPs, RH-NPs, or RM-NPs (C6: 2.5 μg/ml) for 6 hours and then analyzed by flow cytometry (Beckman CytoFLEX, CA, USA). For mitochondrial targeting assessment, cells underwent identical treatment followed by mitochondrial isolation using the protocol described above, with immediate flow cytometry analysis. For confocal microscopy, treated cells were stained with MitoTracker Red (45 min), washed, fixed in 4% PFA, counterstained with DAPI (5 μg/ml, 5 min), and imaged by CLSM.

To investigate the migrasome targeting ability of nanoparticles, 4T1 cells were expanded in 40 cell culture dishes (150-mm diameter). At 60% confluence, cells were stimulated with TL/RH-NPs (equivalent concentration of TPP-LND: 3 μg/ml) and coincubated with C6/RP-NPs, C6/RT-NPs, and C6/RH-NPs (equivalent concentration of C6: 2.5 μg/ml) for 6 or 12 hours. After that, cells were harvested using cell scrapers. A part of the 4T1 cells were used to assess the cellular uptake and mitochondrial of nanoparticles following abovementioned protocols to normalize migrasome uptake of nanoparticles or for comparison. The rest of 4T1 cells were centrifuged at 1000*g* for 10 min to collect the supernatant. This supernatant was then centrifuged at 4000*g* for 10 min, and the resulting supernatant was subjected to final centrifugation at 20,000*g* for 30 min. The pellet was washed once with PBS to obtain the crude migrasome fraction. C6 from harvested cells, mitochondria, and migrasomes were extracted using methanol. After centrifugation (20,000*g*, 10 min), supernatants were analyzed for fluorescence intensity using a microplate reader. The mitochondria and migrasome uptake were normalized to cellular uptake for quantitative comparison. Moreover, to verify the successful extraction of migrasomes, exosomes were extracted in parallel by collecting the culture medium and centrifuging at 300*g* for 10 min and 2000*g* for 30 min. The resulting supernatant was then centrifuged at 100,000*g* for 70 min to get exosomes. Migrasomes, exosomes, and cell body were verified by WB assay using anti-NDST1, anti-Tsg101, and anti-GM130 as the primary antibodies.

### The evaluation of mitocytosis activation

To investigate mitocytosis process across the three breast cancer cell lines, 4T1 cells, E0771 cells, or EMT6 cells were seeded in 12-well plates. When cells grew at 60% confluency, the medium was replaced with serum-free Dulbecco’s modified Eagle’s medium for cell starvation. After 1 hour, 10 μg of *TSPAN4* plasmid was mixed with 250 μl of Opti-MEM and incubated for 5 min, while 10 μl of lipofectamine 3000 transfection reagent was combined with 125 μl of Opti-MEM and incubated separately for 5 min. The plasmid mixture was then added dropwise to the transfection reagent complex and incubated for 15 min. This transfection cocktail was administered to cells for 6 hours, followed by removal and stimulation with TL/RH-NPs (equivalent TPP-LND concentration of 3 μg/ml) for 6 hours. After drug removal, cells on coverslips were stained with MitoTracker (100 nM) at 37°C for 30 min, washed twice with PBS, and imaged by confocal microscopy. For visualizing migrasomes with WGA, 4T1 cells were stained with WGA (20 μg/ml) and Mito Tracker Deep Red (100 nM) and imaged by confocal microscopy.

To assess the mitocytosis activation–triggered TL/RH-NPs by Bio-TEM, 4T1 cells were incubated with TL/RH-NPs, and cells were harvested using cell scrapers and pelleted by centrifugation at 1000 rpm for 5 min. After supernatant removal, the pellet was gently resuspended in 0.5% glutaraldehyde fixative added slowly along the tube wall, followed by overnight fixation at 4°C. Fixed cells were transferred to 1.5-ml microcentrifuge tubes and centrifuged at 12,000 rpm for 10 min, and the supernatant was carefully discarded. The pellet was then overlaid with electron microscopy fixative and stored at 4°C. Following fixation, the cell suspension was transferred to 1.5-ml conical microcentrifuge tubes and centrifuged at 12,000 rpm for 10 min. The supernatant was carefully discarded, and the pellet was preserved. Electron microscopy fixative was slowly added along the tube wall using a pipette, and samples were stored at 4°C. Subsequent processing involved primary fixation with 2.5% glutaraldehyde and postfixation with 1% osmium tetroxide, followed by graded acetone dehydration. Infiltration was performed using acetone/Epon 812 mixtures and then pure Epon 812 embedding. Ultrathin sections (60 to 90 nm) were cut with an ultramicrotome and mounted onto copper grids. Dual staining used uranyl acetate (10 to 15 min) and lead citrate (1 to 2 min) at room temperature prior to TEM imaging.

### In vitro evaluation of mitochondrial damage

Intracellular ROS generation was measured by Reactive Oxygen Species Assay Kit (Beyotime Biotechnology). The 4T1 cells were seeded onto 12-well plates and adhered overnight. Then, the cells were treated with TL/RH-NPs, CGT/RH-NPs, and TL/RH-NPs + CGT/RH-NPs (equivalent concentration of TPP-LND: 3 μg/ml; equivalent concentration of CGT: 3 μg/ml) for 12 hours. Afterward, the culture medium containing drugs was discarded, and the cells were further incubated with 20,70-dichlorofluorescein diacetate for 25 min. Last, the flow cytometer (FACS Calibur, BD, USA) was used to quantify the generation of ROS.

Inhibition of nanoparticles on mitochondrial respiration complex II was measured by mitochondrial respiration complex II assay kit (Beyotime Biotechnology). The 4T1 cells were seeded onto 12-well plates and adhered overnight. Then, the cells were treated with TL/RH-NPs, CGT/RH-NPs, and TL/RH-NPs + CGT/RH-NPs (equivalent concentration of TPP-LND: 3 μg/ml; equivalent concentration of CGT: 3 μg/ml) for 12 hours. The cells were collected, and the mitochondrial complex II enzyme-linked immunosorbent assay kit was used for detection.

The mitochondrial membrane potential (Δψm) was evaluated by Mitochondrial Membrane Potential Assay Kit with JC-1 (Beyotime Biotechnology). Briefly, 4T1 cells were seeded onto 12-well plates overnight, followed by treatment with TL/RH-NPs, CGT/RH-NPs, and TL/RH-NPs + CGT/RH-NPs (equivalent concentration of TPP-LND: 3 μg/ml; equivalent concentration of CGT: 3 μg/ml) for 12 hours. Afterward, the cells were harvested and stained with JC-1 staining working solution for 15 min. The fluorescence intensity of each group was detected by flow cytometry.

To investigate mitochondrial morphological alterations, 4T1 cells were seeded in medium-sized culturing dishes and incubated with TL/RH-NPs and Combo for 12 hours. After incubation, medium was aspirated, and cells were washed thrice with PBS. Cells were harvested using scrapers and fixed with 2.5% (w/v) glutaraldehyde at 4°C for 24 hours, followed by postfixation with 1% (w/v) osmium tetroxide (OsO_4_) for 2 hours at room temperature. Specimens were then dehydrated through a graded ethanol series, infiltrated overnight with Embed 812 resin, and polymerized at 60°C for 48 hours. Ultrathin sections (70 nm) were stained with 2% (w/v) uranyl acetate and lead citrate prior to imaging via TEM.

### In vitro evaluation of antimetastatic efficiency

The potential inhibitory effects of nanoparticles on migration were investigated using wound healing assay. 4T1 cells were seeded onto 24-well plates and adhered overnight before using a 200-μl pipet tip to create scratch wounds. Afterward, the culture medium containing TL/RH-NPs, CGT/RH-NPs, and TL/RH-NPs + CGT/RH-NPs (Combo) (equivalent TPP-LND and CGT doses of 3 μg/ml) or a different ratio of TPP-LND:CGT or CGT, CGT/RP-NPs, CGT/RT-NPs, and CGT/RH-NPs were added into the corresponding wells to treat cells for 24 hours. Then, the images of wound healing were captured by a microscope (IX81, Olympus, Japan) and analyzed by ImageJ software.

The potential inhibitory effects of nanoparticles on migration and invasion were further investigated using transwell inserts (8 μm, Corning, USA). 4T1 cells in 100 μl of incomplete 1640 medium were added to the upper chamber for 4 hours, with 600 μl of cell-free complete 1640 medium added to the down chamber. After that, TL/RH-NPs, CGT/RH-NPs, and TL/RH-NPs + CGT/RH-NPs (Combo) (equivalent TPP-LND and CGT doses of 3 μg/ml) on the upper chamber were coincubated with 4T1 cells for 24 hours. After incubation, cells were fixed with formalin, stained with 0.2% crystal violet, and washed with PBS three times. Then, the cells in the upper chamber were wiped carefully and photographed with a microscope (Leica Microsystems, Germany). Last, the crystal violet was eluted by 33% acetic acid eluent and measured with a Varioskan Flash 902-ULTS (optical density at 590 nm, Thermo Fisher Scientific, USA). Moreover, the in vitro invasion assay was carried out in a manner that was similar to the migration assay, but the inner bottom of the chamber was precoated with Matrigel (50 μg/ml; Corning, USA) before seeding of 4T1 cells.

To investigate the effects of migrasomes in cell migration and its influence on the antimetastatic effects of TL/RH-NPs, reverse transcription quantitative polymerase chain reaction (RT-qPCR) was first adopted to verify the silence efficiency of constructed si-TSPAN4. The small interfering RNAs were transfected into 4T1 cells by lipofectamine 3000 according to the manufacturer’s protocol. The si-TSPAN4 (2.5 μg) or negative control RNA were incubated with 4T1 cells for 6 hours. For qRT-PCR, the cells were harvested 48 hours after transfection of si-TSPAN4 in vitro. The total RNA in cells was extracted according to the method of the M5 Universal RNA Mini Kit. The primers for *TSPAN4* and *GAPDH* were presented in the “Materials and reagents” section. The cycling program was 95°C for 15 s, 95°C for 5 s, and 60°C for 30 s (40 cycles). The relative level of RNA was computed using the 2^−ΔΔCt^ analysis method. Migration assay was carried out following the steps mentioned above by seeding cells transfected with negative control, si-TSPAN4 silenced 4T1 cells, or si-TSPAN4 silenced 4T1 cells treated with TL/RH-NPs in the upper chamber.

To demonstrate the interactions between integrin α5 and CGT, we compared the effects of different CGT formulations on integrin α5 expression. 4T1 cells were seeded onto 24-well plates and incubated for 12 hours. After that, cells were treated with TL/RH-NPs to trigger mitocytosis and further treated with CGT, CGT/RT-NPs, CGT/RH-NPs, and Combo (equivalent TPP-LND and CGT doses of 3 μg/ml). After 12 hours, cells were collected and incubated with anti-integrin α5–FITC antibody for flow cytometry.

### In vitro inhibition of mitocytosis

To evaluate the capacity of nanoparticles to suppress mitocytosis, 4T1 cells were seeded in six-well plates for 24 hours, followed by 12 hours of incubation with CCCP, TL/RH-NPs, CGT/RH-NPs, and Combo (equivalent concentrations of CCCP: 2 μg/ml; CGT: 3 μg/ml; and TPP-LND: 3 μg/ml). Cells were harvested via scrapers and lysed in RIPA buffer containing protease/phosphatase inhibitors. After centrifugation at 10,000*g* for 10 min, supernatants were mixed with RIPA buffer, boiled (15 min), and subjected to WB using primary antibodies against KIF5B (1:1000 dilution) and α-tubulin (1:1000 dilution). To further investigate the induction of mitophagy, 4T1 cells were seeded in six-well plates for 24 hours, followed by 12 hours of incubation with TL/RH-NPs (equivalent concentration of TPP-LND: 6 μg/ml) and TL/RH-NPs (equivalent concentration of TPP-LND: 3 μg/ml). Cells were harvested and mixed with RIPA buffer, boiled (15 min), and subjected to WB using primary antibodies against LC-3B (1:1000 dilution) and p62 (1:1000 dilution).

To visualize migrasome expression via confocal microscopy, cells were transfected with migrasome-specific marker proteins TSPAN4 following the protocol detailed in the evaluation of mitocytosis activation. Transfected cells were stimulated for 6 hours with TL/RH-NPs, CGT/RH-NPs, and Combo (equivalent concentration of CGT: 3 μg/ml; TPP-LND: 3 μg/ml). After incubation, cells were washed twice with PBS. Glass coverslips were mounted and imaged using confocal microscopy.

To investigate mitochondrial transfer between cells in mitocytosis process, donor 4T1 cells were seeded in 12-well plates for 24 hours and incubated with C6/RH-NPs (equivalent dose of C6: 2.5 μg/ml) and TL/RH-NPs (equivalent dose of TPP-LND: 3 μg/ml) for 6 hours. TL/RH-NPs induced mitochondrial damage and activated mitocytosis, while C6/RH-NPs labeled impaired mitochondria. After replacing the medium to initiate spontaneous exocytosis (simulating mitocytosis-mediated expulsion), C6-marked mitochondria entered migrasomes and were expelled into the medium within 3 hours. Conditioned medium containing migrasomes was collected and supplemented with TL/RH-NPs, CGT/RH-NPs, or Combo (equivalent dose of TPP-LND: 3 μg/ml and equivalent dose of CGT: 3 μg/ml). Healthy recipient 4T1 cells were exposed to this medium for 3 hours. Uptake of mitochondria-laden migrasomes was quantified to evaluate nanoparticle effects on mitocytosis-mediated intercellular transfer. Controls received C6/RH-NPs alone with identical processing.

### In vivo evaluation of therapeutic efficiency

In antitumor and antimetastasis evaluation of TL/RH-NPs, an orthotopic breast cancer mouse model was established by implanting 4T1 cells into one mammary fat pad of female Balb/c mice. An orthotopic breast cancer mouse model was established by implanting E0771 cells into one mammary fat pad of female C57 mice. An orthotopic breast cancer mouse model was established by implanting EMT6 cells into one mammary fat pad of female Balb/c mice. Once the tumor volume reached ~200 mm^3^, tumor-bearing mice were randomly divided into four groups (*n* = 5). These groups were then intravenously administered with different treatments, including saline, TL/T-NPs, TL/RT-NPs, and TL/RH-NPs. All groups received a total of five doses administrated every other day (equivalent dose of TPP-LND: 2 mg/kg).

In antitumor evaluation of Combo, an orthotopic breast cancer mouse model was established by implanting 4T1 cells into one mammary fat pad of female Balb/c mice. Once the tumor volume reached ~200 mm^3^, tumor-bearing mice were randomly divided into six groups (*n* = 8). These groups were then intravenously administered with different treatments, including saline, TL/RH-NPs, CGT/RH-NPs, TL/RP-NPs + CGT/RP-NPs, TL/T-NPs + CGT/T-NPs, and TL/RH-NPs + CGT/RH-NPs (Combo-200). Moreover, once the tumor volume reached ~50 mm^3^, the 4T1 tumor–bearing Balb/c mice were then intravenously administered with TL/RH-NPs + CGT/RH-NPs (Combo-50) (*n* = 8). All groups received a total of five doses administrated every other day (equivalent TPP-LND dose of 2 mg/kg and equivalent CGT dose of 2 mg/kg).

When the control group reached 1500 mm^3^, the mice were euthanized, and tumors were collected. The tumor inhibition rate was calculated by the following formula: tumor inhibition rate (%) = (tumor weight of saline group − tumor weight of therapy group)/(tumor weight of saline group) × 100%. During this process, the tumor volume and body weight were monitored every day.

The therapeutic efficacy of each group was evaluated by closely monitoring tumor size every day. Tumor volume was calculated using the formula: tumor volume (mm^3^) = *W*^2^ × *L*/2, where *L* represents the larger tumor diameter and *W* represents the smaller tumor diameter.

To assess the antimetastatic effects, mice from each group were euthanized, and tumors were excised. Lungs were subsequently submerged in Bouin’s fluid overnight, and the number of pulmonary metastatic nodules was counted by three different researchers. In addition, tumors and excised organs were fixed in 4% PFA and embedded in paraffin for further analysis. A disseminated lung metastatic Balb/c mice model was developed by inoculating 1 × 10^6^ 4T1-Luc cells intravenously to assess the ability of Combo to prevent tumor hematogenous metastasis. Mice were subsequently randomly assigned to three groups and treated with saline, TL/RP-NPs + CGT/RP-NPs, and TL/RH-NPs + CGT/RH-NPs (Combo). Bioluminescence images were taken using an IVIS Spectrum Imaging System (PerkinElmer Ltd.) on days 4 and 10 to assess the lung metastatic progression of 4T1-Luc, and data were analyzed by PerkinElmer Ltd.’s Living Image software after 10 min following intraperitoneal administration of d-luciferin (15 mg/kg) into the animals.

To assess the survival of 4T1 tumor–bearing Balb/c mice treated with Combo, an orthotopic breast cancer mouse model was established by implanting 4T1 cells into one mammary fat pad of female Balb/c mice. Once the tumor volume reached ~200 mm^3^, tumor-bearing mice were randomly divided into six groups (*n* = 8). These groups were then intravenously administered with different treatments, including saline, TL/RH-NPs, CGT/RH-NPs, TL/RP-NPs + CGT/RP-NPs, TL/T-NPs + CGT/T-NPs, and TL/RH-NPs + CGT/RH-NPs (Combo-200) (equivalent TPP-LND dose of 2 mg/kg and equivalent CGT dose of 2 mg/kg). Tumor size and animal survival were recorded every other day until 27 days.

### Statistical analysis

The results were expressed as means ± standard deviation. For statistical analysis between two groups, Student’s *t* test for independent means was applied. Comparisons between multiple groups were made by one-way analysis of variance (ANOVA). Statistical analysis was performed using the SPSS 22.0 software (SPSS Inc., USA). A value of *P* < 0.05 was considered as statistically significant.
