# Supplementary material for: Targeting mitocytosis potentiates mitochondria drug delivery for antimetastasis therapy
Source: Sci Adv. 2026 Apr 10;12(15):eaec7150. doi: 10.1126/sciadv.aec7150 (PMC13068050; doi:10.1126/sciadv.aec7150)
Supplement: Supplementary file 1 — Figs. S1 to S11 [file sciadv.aec7150_sm.pdf]

Science Advances
AAAS

Supplementary Materials for

Targeting mitocytosis potentiates mitochondria drug delivery for antimetastasis therapy

Yudi Deng *et al.*

Corresponding author: Lian Li, liliantripple@163.com; Yuan Huang, huangyuan0@163.com

Sci. Adv. **12**, eaec7150 (2026)
DOI: 10.1126/sciadv.aec7150

This PDF file includes:

Figs. S1 to S11

fig. S1.

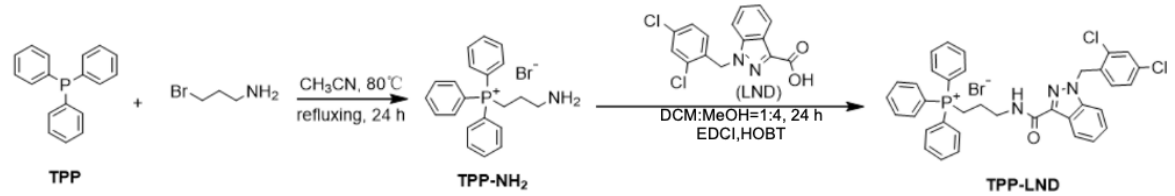

fig. S1. The synthetic routine of TPP-LND.

fig. S2.

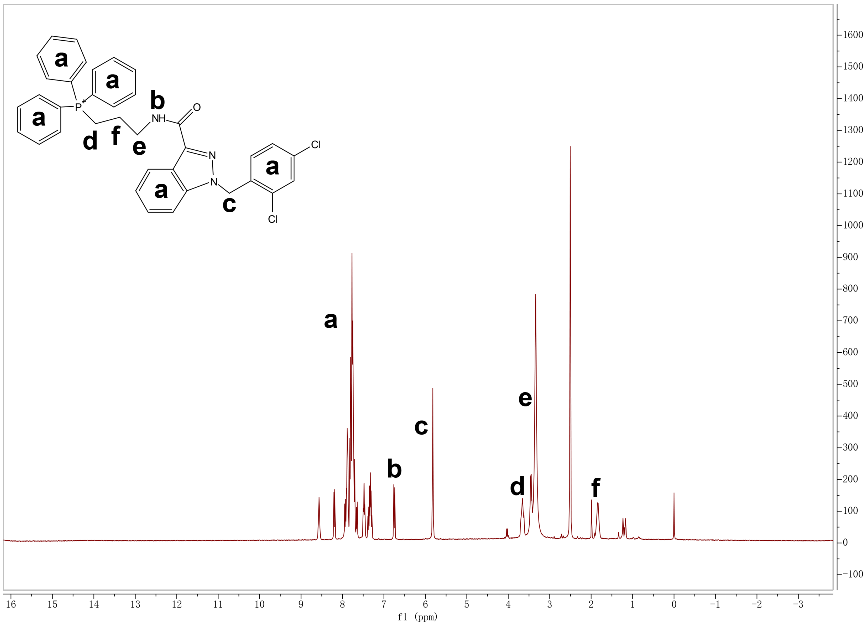

fig. S2. The ^{1}H-NMR spectrum of TPP-LND. The ^{1}H NMR data for TL was as follows:^{1}H NMR (400 MHz, DMSO-d6) δ 8.56 (t, J = 6.0 Hz, 1H), 8.20 (d, J = 8.2 Hz, 1H), 7.91-7.85 (m, 3H), 7.82-7.62 (m, 14H), 7.48 (td, J = 8.5, 7.8, 2.8 Hz, 1H), 7.41-7.27 (m, 2H), 6.75 (d, J = 8.4 Hz, 1H), 5.82 (s, 2H), 3.66 (dt, J = 14.6, 7.4 Hz, 2H), 3.45 (q, J = 6.8 Hz, 2H), 1.77-1.92 (m, 2H)。

fig. S3.

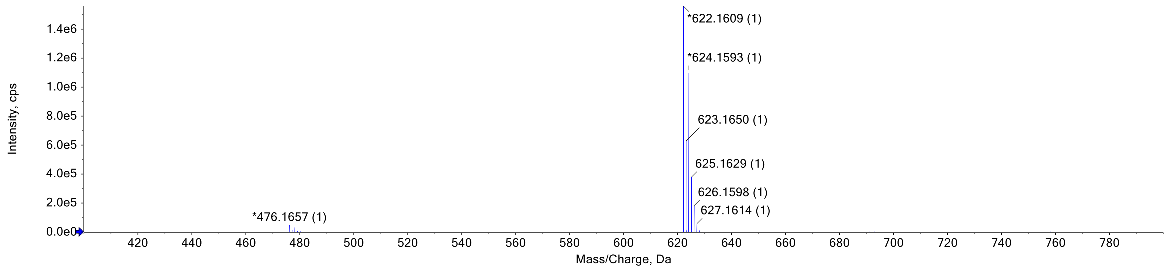

fig. S3. The mass spectrum of TPP-LND. The structure of TPP-LND was analyzed by mass spectrometry.

fig. S4.

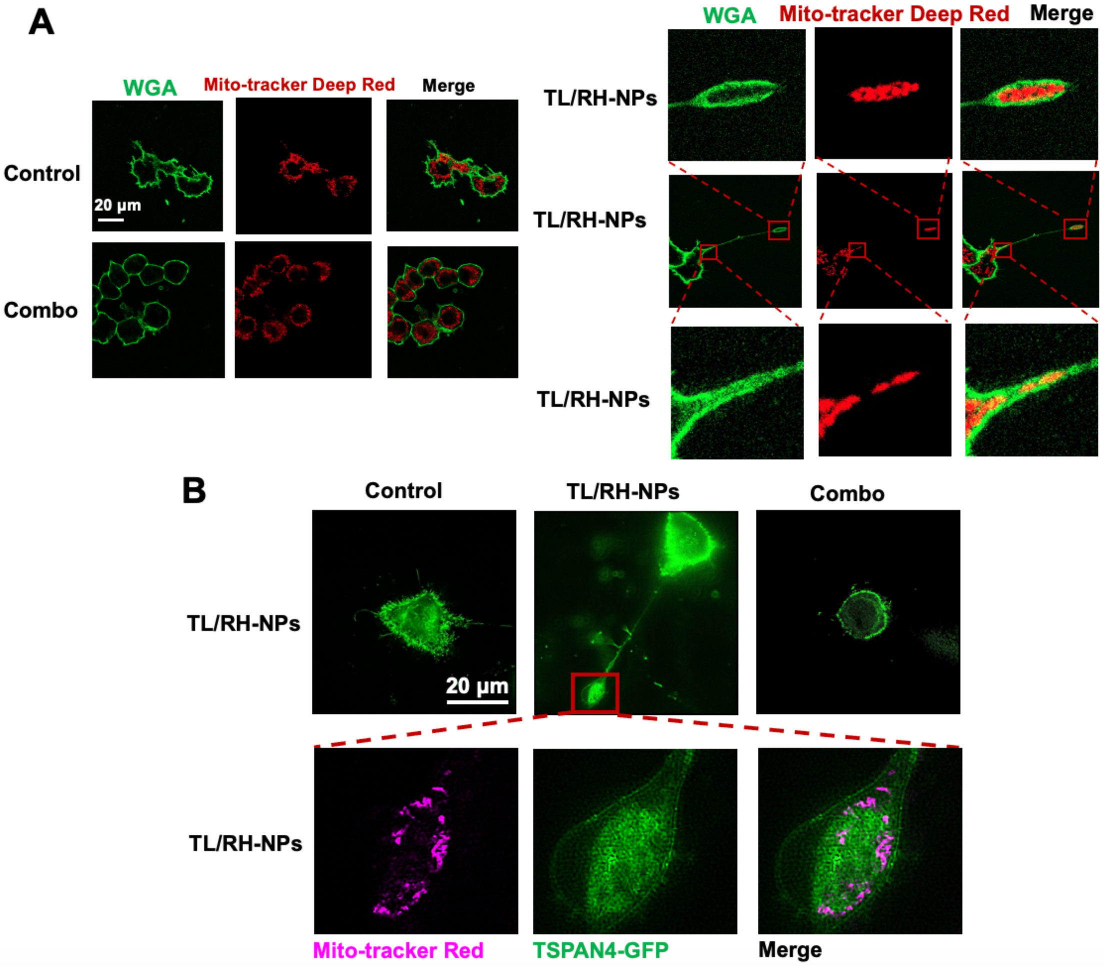

fig. S4. Laser con-focal images of mitocytosis activation. (**A**) 4T1 cells are stained with WGA and mitochondria are marked by Mito-tracker Deep Red after treated with TL/RH-NPs or Combo. (**B**) 4T1 cells are transfected with migrasome characteristic protein TSPAN4 (green) and mitochondria are marked by Mito-tracker Deep Red after treated with TL/RH-NPs or Combo.

fig. S5.

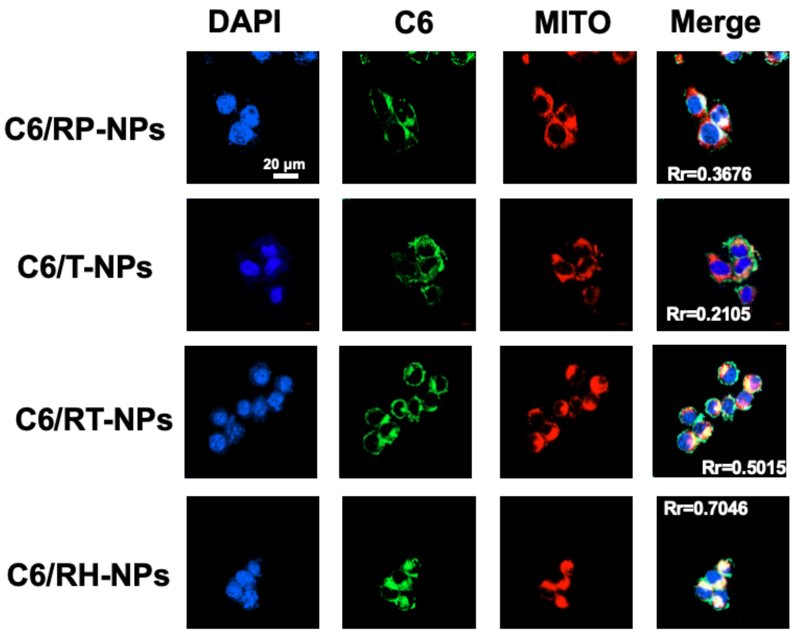

fig. S5. Mitochondria targeting ability of nanoparticles. Con-focal images of C6/RP-NPs、C6/T-NPs、C6/RT-NPs and C6/RH-NPs for mitochondrial distribution at 6 h.

fig. S6.

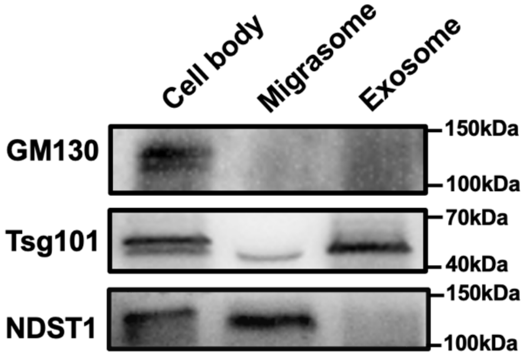

fig. S6. Samples from cell bodies, migrasomes and exosomes were analyzed by western blotting using antibodies against GM130 (marker protein for cell body), Tsg101 (marker protein for exosome) and NDST1 (marker protein for migrasome).

fig. S7.

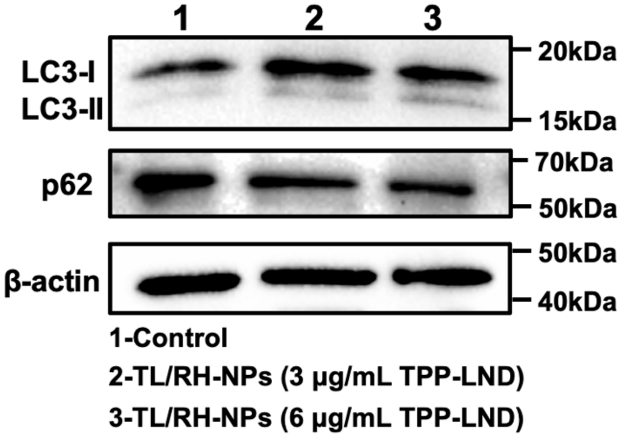

fig. S7. WB analysis of the autophagic substrate (LC3B) and p62 expression in 4T1 cell treated with different concentrations of TL/RH-NPs.

fig. S8.

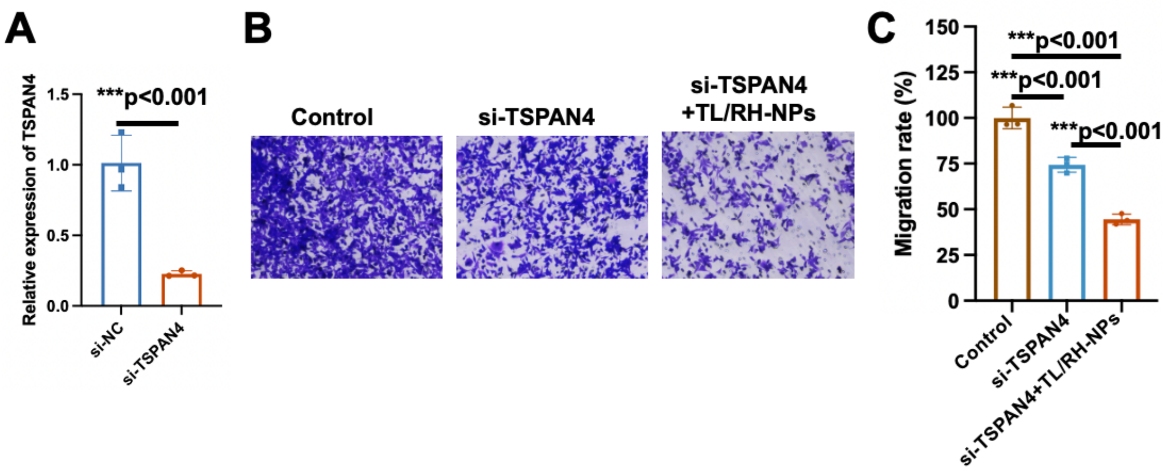

fig. S8. (A) The knockdown efficiencies for TSPAN4 treated with si-TSPAN4 or negative control RNA (si-NC). (**B**) Representative microscopy images of the migrated 4T1 cells and (**C**) statistical analysis of the migration rate of 4T1 cells treated with si-TSPAN4 or si-TSPAN4 plus TL/RH-NPs for 24 h. All results were presented as mean ± SD, n=3.

fig. S9.

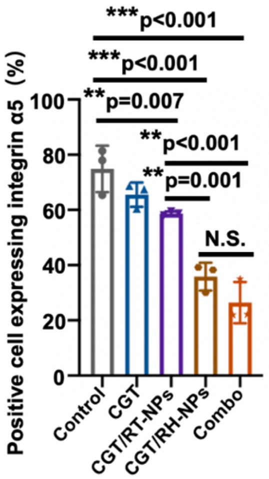

fig. S9. Positive cell expressing integrin α5 after treated with CGT, CGT/RT-NPs, CGT/RH-NPs and Combo. All groups were pre-treated with TL/RH-NPs. All results were presented as mean ± SD, n=3. NS indicated no significance.

fig. S10.

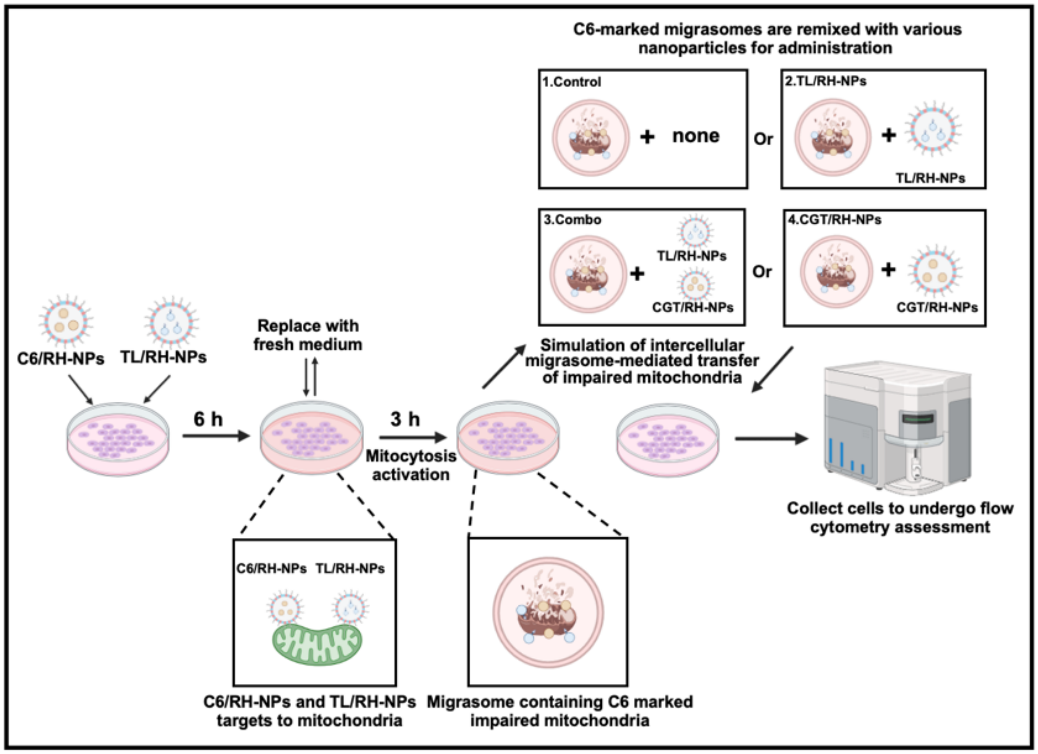

fig. S10. Illustration of intracellular transport of damaged mitochondria between 4T1 cells.

fig. S11.

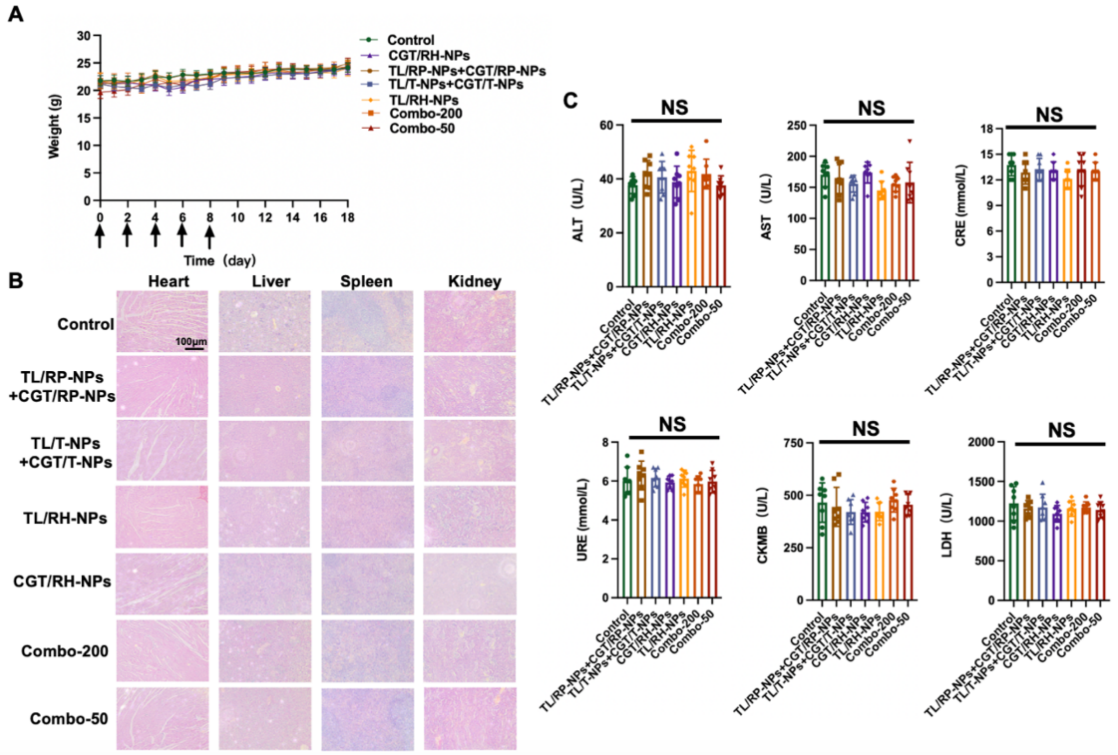

fig. S11. Biosafety of nanoparticles. (A) Body weight of 4T1 tumor bearing Balb/c mice during treatment of saline, CGT/RH-NPs, TL/RP-NPs+CGT/RP-NPs, TL/T-NPs+CGT/T-NPs, TL/RH-NPs, Combo-200 and Combo-50. **(B)** H&E staining images of major organs from mice of saline, CGT/RH-NPs, TL/RP-NPs+CGT/RP-NPs, TL/T-NPs+CGT/T-NPs, TL/RH-NPs, Combo-200 and Combo-50. **(C)** Blood biochemical assessment of ALT, AST, CRE,URE, CKMB and LDH. All results were presented as mean ± SD, n=8. NS indicated no significance.
